# Analysing the distance decay of community similarity in river networks using Bayesian methods

**DOI:** 10.1038/s41598-021-01149-x

**Published:** 2021-11-04

**Authors:** Filipe S. Dias, Michael Betancourt, Patricia María Rodríguez-González, Luís Borda-de-Água

**Affiliations:** 1 CIBIO/InBio, Centro de Investigação em Biodiversidade e Recursos Genéticos, Laboratório Associado, Universidade do Porto, Campus Agrário de Vairão, 4485-661 Vairão, Portugal; 2 CIBIO/InBio, Centro de Investigação em Biodiversidade e Recursos Genéticos, Laboratório Associado, Instituto Superior de Agronomia, Universidade de Lisboa, Tapada da Ajuda, 1349-017 Lisbon, Portugal; 3 BIOPOLIS Program in Genomics, Biodiversity and Land Planning, CIBIO, Campus de Vairão, 4485-661 Vairão, Portugal; 4 Symplectomorphic, LLC., New York, USA; 5 Centro de Estudos Florestais, Instituto Superior de Agronomia, Universidade de Lisboa, Tapada da Ajuda, 1349-017 Lisbon, Portugal

**Keywords:** Ecology, Biodiversity, Community ecology, Ecological modelling, Ecosystem ecology, Forest ecology, Freshwater ecology, Riparian ecology

## Abstract

The distance decay of community similarity (DDCS) is a pattern that is widely observed in terrestrial and aquatic environments. Niche-based theories argue that species are sorted in space according to their ability to adapt to new environmental conditions. The ecological neutral theory argues that community similarity decays due to ecological drift. The continuum hypothesis provides an intermediate perspective between niche-based theories and the neutral theory, arguing that niche and neutral factors are at the opposite ends of a continuum that ranges from competitive to stochastic exclusion. We assessed the association between niche-based and neutral factors and changes in community similarity measured by Sorensen’s index in riparian plant communities. We assessed the importance of neutral processes using network distances and flow connection and of niche-based processes using Strahler order differences and precipitation differences. We used a hierarchical Bayesian approach to determine which perspective is best supported by the results. We used dataset composed of 338 vegetation censuses from eleven river basins in continental Portugal. We observed that changes in Sorensen indices were associated with network distance, flow connection, Strahler order difference and precipitation difference but to different degrees. The results suggest that community similarity changes are associated with environmental and neutral factors, supporting the continuum hypothesis.

## Introduction

The distance decay of community similarity (DDCS) states that geographically close communities tend to be more similar than those that are further apart^1,2^. The DDCS is implicit in several ecological phenomena such as species turnover along environmental gradients^3^, source-sink dynamics^4,5^, metapopulations^6^, and the theory of island biogeography^7^. There are three major perspectives or hypothesis for explaining this ecological pattern^2,8,9^. Niche-based theories argue that as environmental conditions change, species are sorted according to their ability to adapt to new conditions and habitats^10,11^. The ecological neutral theory^12^ argues that community similarity decays due to ecological stochasticity, caused by random births and deaths in a population (i.e., ecological drift)^13^, random dispersal, and dispersal limitation. The continuum hypothesis provides an intermediate perspective, arguing that niche and neutral factors are, in fact, at the opposite ends of a continuum that ranges from competitive exclusion to stochastic exclusion^14^. Understanding the ecological mechanisms that underpin the DDCS is crucial for analysing changes in community composition and for correctly identifying the impacts of anthropogenic activities, climate change and for developing effective conservation plans^15,16^.

The first studies on the distance decay of community similarity date from the 1960s but it was Nekola and White^1^ who laid the foundations for using distance decay rates to describe, compare, and understand biodiversity patterns^17^. The authors examined distance decay rates in vascular plants and bryophytes of boreal and montane spruce-fir forests in North America and observed negative exponential decay rates. More importantly, they observed that decay rates varied between vascular plants and bryophytes, with growth forms, between dispersal methods, and at different scales. Subsequent studies built on this framework by adding the analysis of how changes in environmental conditions affect community similarity^2,18,19^.

Riverine ecosystems support extremely high levels of biodiversity and provide key ecosystem services^20,21^. So far, few studies analysed the DDCS in riparian plant communities and the results are disparate. For instance, Rouquette et al.^22^ studied an urban river network in the UK and found no significant association between Euclidean, network and flow distance and changes in plant community similarity after accounting for environmental differences. In Toronto, Canada, Kuglerová et al.^23^ studied riparian vegetation in seven river basins and found that after accounting for environmental differences, the distance was only a significant predictor for community composition changes in three out of seven basins. Finally, in China, Zhang et al.^24^ studied aquatic macrophytes in a large river basin and concluded that community similarity changes were significantly associated with distance, even after accounting for environmental covariates. Despite these studies, there is still little data on the relative importance of niche-based factors and neutral factors, which are crucial to understand structure and community assembly processes in riverine ecosystems.

In this study, we analysed the factors that are associated with the DDCS in riparian plant communities. Specifically, we aimed to assess the effect of niche and neutral factors on community similarity as measured by the Sorensen’s index^25^. We selected two covariates that we considered predominately neutral (network distance and flow connection) and two covariates that we considered predominately niche-based (precipitation difference and Strahler order difference). We ensured that both neutral and niche-based covariates had low pairwise correlations. Despite the careful selection process, the neutral/niche-based covariates may be correlated with unmeasured niche-based factors/neutral covariates, therefore we cannot assume a one to one relationship between each covariate and the corresponding factors. We fit a model with these four covariates and assessed the relative support for the neutral theory, niche-based theories and the continuum hypothesis based on the association between the covariates and Sorensen indices. We expected to find that both neutral and niche-based would to some extent be associated with changes in community similarity.

## Methods

### Study area

**Figure 1:**
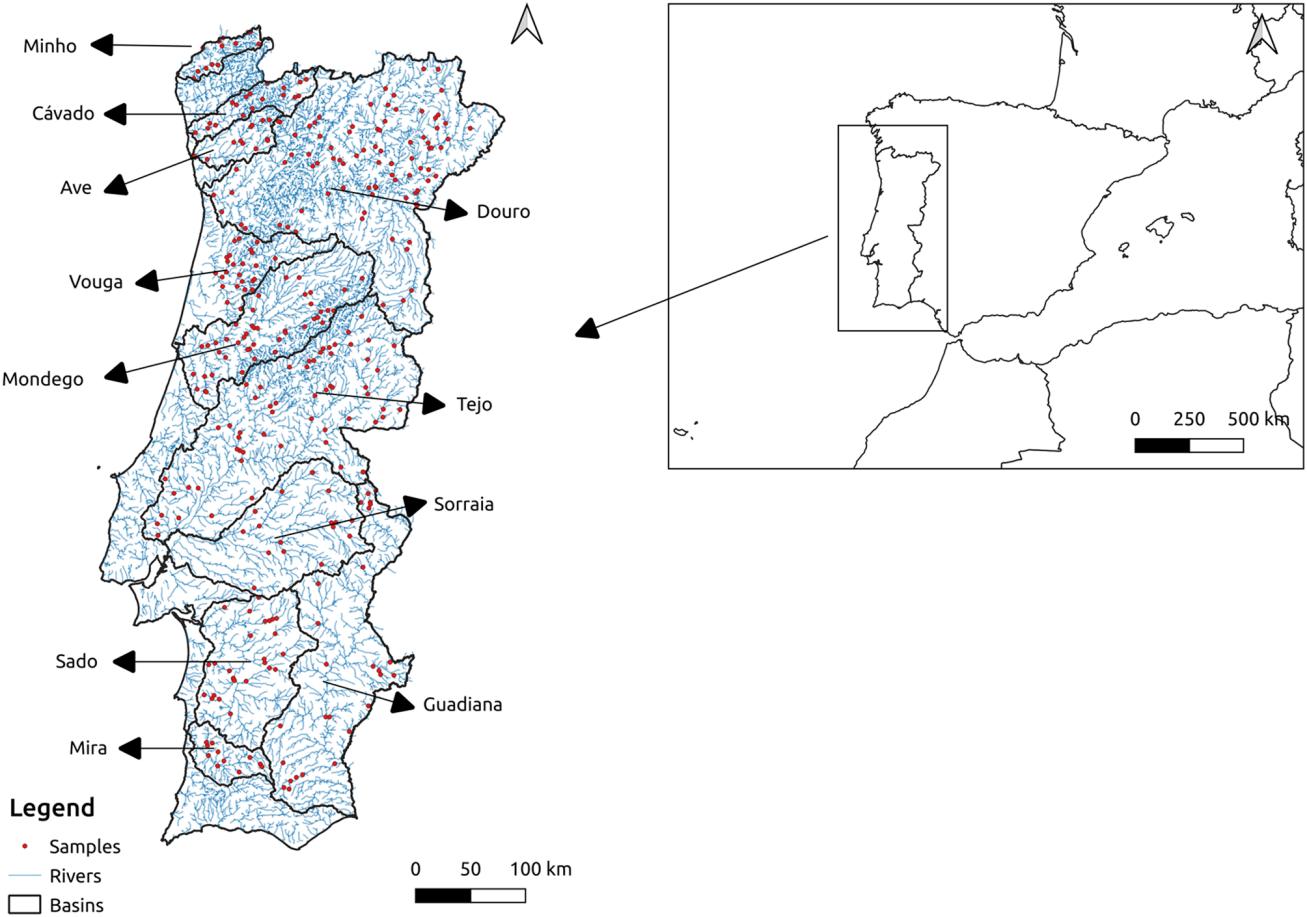
Continental Portugal and the location of the vegetation plots in the eleven river basins studied (red dots). This figure was created with QGIS 3.20.3^63^.

This study took place in mainland Portugal, southwestern Europe, between 37° and 42°N (Fig. 1). The northern half is hilly, with 95% of the area above 400 m, while the south is flatter, with 62% of the area below 200 m. The climate ranges between Temperate in the North and Mediterranean in central and southern Portugal and presents a significant climatic and altitudinal gradient^26^. In general, temperature increases and precipitation decreases when moving from north to south and west to east. Mean annual temperatures range between 7 °C in the mountains of central Portugal and 18 °C in the southern coastal region. The highest mean annual precipitation occurs along the highlands in the northwestern region (> 3000 mm/year) and the lowest along the southern coast and the eastern part of the territory (below or around 500 mm). On average, about 42% of the annual precipitation falls during the winter season (December–February), and only 6% during Summer (June–August)^27^.

### Data collection and processing

The vegetation data were collected between 2003 and 2006 in 404 sites across 38 river basins in continental Portugal during the pre-assessment surveys conducted to implement the Water Framework Directive (WFD)^28–30^. Field surveys followed the protocol defined for the WFD implementation^29^, which involved establishing a 100-m plot along the fluvial corridor with a width corresponding to regular floods in each site and all plant species were identified. In smaller rivers plot sizes were smaller and in larger rivers plot sizes were larger, which ensured we obtained a representative sample of the community. Plots were evenly distributed across the river basin to maximize coverage. No plant specimens were collected or damaged during fieldwork.

For this study, we selected the 11 largest river basins, which were the ones for which there were at least ten vegetation surveys. For the remaining 26 river basins, there were only 3–5 surveys each which we considered insufficient for carrying out this study.

We calculated the Sorensen similarity index between pairs of sites within the same river basin with:

$$ {\text{Sorensen index }} = 2{\text{a}}/2{\text{a}} + {\text{b}} + {\text{c}} $$

where *a* is the number of species in common between both sites, *b* is the number of unique species on the first site, and *c* is the number of unique species on the second site. Therefore, the Sorensen index is the number of species in common between two sites divided by their average number of species. An index value of 1 indicates that communities have the same species composition, while 0 indicates that communities share zero species. We ran calculations with the package “vegan 2.57”^31^ using R 4.0.4^32^.

We followed a careful selection process for choosing neutral and niche-based covariates (Table 1). We started by considering differences in distance (Euclidean, network and flow), in climate variables (e.g., mean, maximum and minimum precipitation and temperature), in geomorphological variables (e.g. altitude, slope, aspect) and in land use variables (e.g. forest, shrublands). We visually selected covariates with clear positive or negative relationships with Sorensen indices and then chose a subset of covariates with low pairwise correlations (< 0.27).

**Table 1:** List of neutral and niche-based variables included in the model developed to assess the relative importance of both neutral and niche-based factors for explaining changes in community similarity measured by Sorensen’s index.

Classification	Variable	Description and units
Neutral	Network distance	Network distance between a pair of vegetation samples (km)
	Flow connection	1—samples are flow connected, 0—samples are not flow connected
Niche-based	Strahler order difference	Strahler order difference between the pair of vegetation samples
	Precipitation difference	Difference in annual precipitation between the pair of vegetation samples

**Figure 2:**
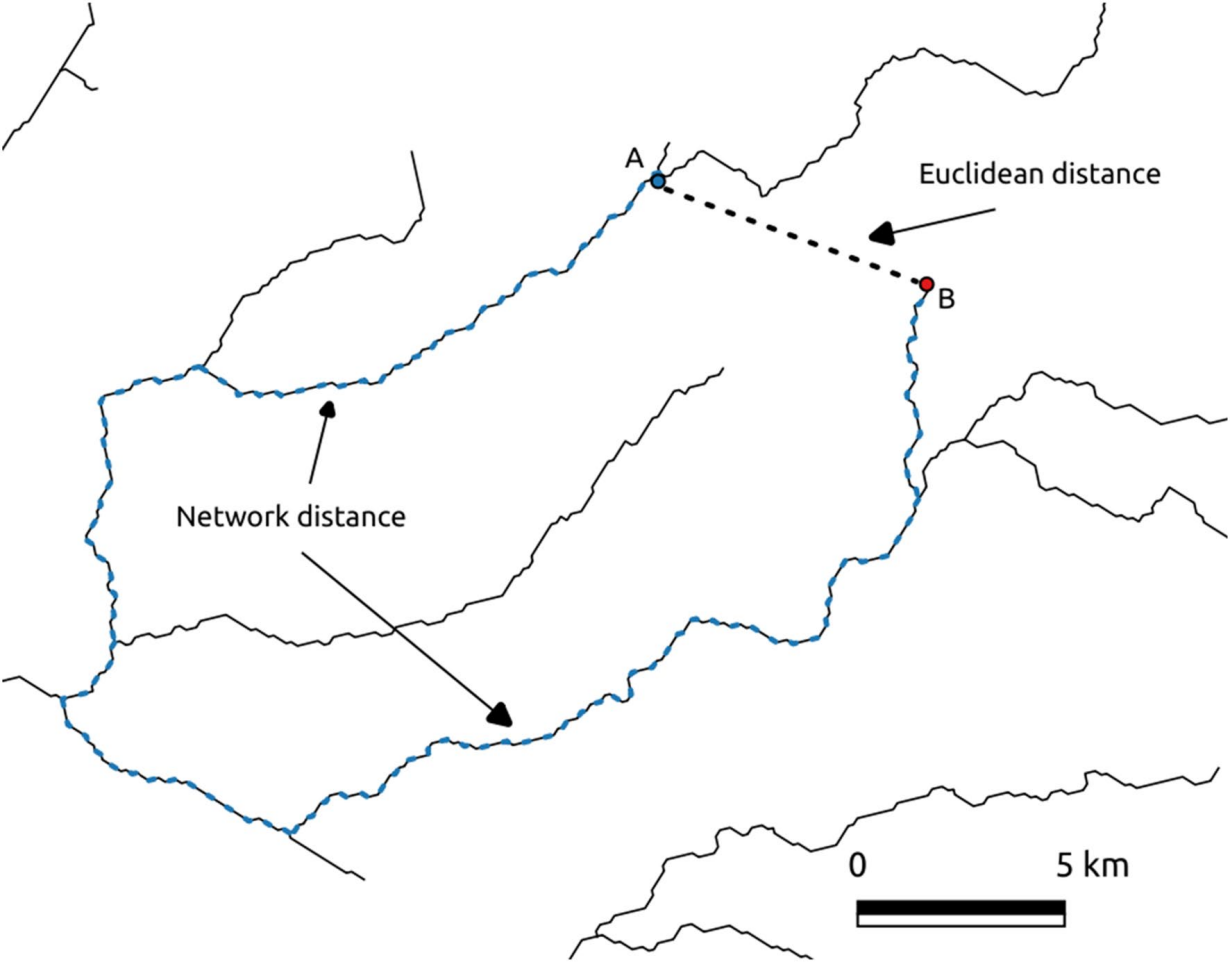
This figure shows two riparian vegetation samples, A and B (blue and red dot) that are separated by a large network distance (dashed blue line) and by a small Euclidean distance (dashed black line).

As neutral covariates, we selected the network distance and flow connection. The concept of distance deserves some considerations. In terrestrial ecosystems, scientists usually measure the distances between communities using the Euclidean distance which is the straight line distance between two sites. However, in riverine ecosystems habitats, the Euclidean distance does not adequately account for the spatial configuration, connectivity, directionality and relative location of the sites in the river network^22,33^. An alternative is to measure the network distance, which is the distance between two sites along the river network. However, there is a caveat concerning the use of network distances. Two geographically close sites can be separated by a large network distance. If we look at Fig. 2 we see that sites A and B are relatively close as judged by the Euclidean distance but very far when considering the network distance. Given that riparian plant species’ seeds can be dispersed by several means other than water (e.g., wind and animals) we can expect sites A and B to share a higher proportion of species than we would expect if we just considered the network distance. In order to isolate the effect of network distance on community similarity we excluded pairs of sites for which the ratio between the network distance and the Euclidean distance was equal or lower than 2. As such, the number of unique site pairs was reduced from 7846 to 3857. In addition to network distances, we also calculated the covariate flow connection, which is a binary variable that denotes whether two vegetation samples are connected by flow (1) or not (0). We calculated the network distance and flow connection with the R package “igraph 1.26”^34^ and “shp2graph 0-5”^35^. We used river network from CCM River and Catchment Database Version 2.0^36^.

As niche-based covariates we selected two variables, differences in annual precipitation and differences in Strahler order. Annual precipitation is a good proxy for relative humidity, hydrological regime, and potential evapotranspiration. These are good predictors for riparian plant communities' composition in Mediterranean environments^20^. We calculated the absolute value of the difference in mean annual precipitation between two sites using data between 1960 and 1990^37^. We also included the Strahler order, a measure of network position and stream size. We consider the Strahler order a niche variable because streams with lower Strahler order tend to be shallower, narrower, and closer to the river source. In comparison, streams with higher Strahler order are deeper, broader, and closer to the river mouth, thus denoting several environmental characteristics of the riverine ecosystem^21,38^. We calculated the absolute value of the difference in Strahler order using Strahler order values provided by the CCM River and Catchment Database Version 2.0^36^.

### Data analysis

To analyse the data, we used a hierarchical Bayesian approach^39^. We begin by introducing the model’s formula and then provide a more detailed explanation.

#### Likelihood

$$ {\text{Sorensen index }}\sim {\text{ Beta distribution }}\left( {\mu ,\kappa } \right) .$$

$$\begin{aligned}{\text{logit}}\left( \upmu  \right) & = {\upalpha _{{\text{baseline}}}} + {\upalpha _{\text{s}}}_{\left[ {{\text{comm}}.1} \right]} + {\upalpha _{\text{s}}}_{\left[ {{\text{comm}}{{.2}}} \right]} + {\upalpha _{\text{c}}}_{\left[ {basin} \right]} + {\upalpha _{\text{o}}}_{\left[ {{\text{strahler}}} \right]}\\ & \quad  + \upbeta {1_{\left[ {basin} \right]}} \, {\text{Network}}\;{\text{distance}} + \upbeta {2_{\left[ {basin} \right]}} \, {\text{Flow}}\;{\text{connection}} + \upbeta {3_{\left[ {basin} \right]}} \, {\text{Precipitation}}\;{\text{difference}}\end{aligned}$$

#### Priors

- α _baseline_ ~ Normal (0,0.3)
- *α*_*s*_ ~ Normal (0,*σ*_*s*_)
- *σ*_*s*_ ~ Exponential(1)
- *α*_*c*_ ~ Normal (0,0.3)
- *α*_*o*_ ~ Normal (0,*σ*_*Strahler*_)
- *σ*_*Strahler*_ ~ Exponential (2)
- *β*1_[*basin*]_ ~ Normal (*μ*_distance_, *σ*_distance_)
- *β*2_[*basin*]_ ~ Normal (*μ*_*flow*_, *σ*_*flow*_)
- *β*2_[*basin*]_ ~ Normal (*μ*_precipitation_, *σ*_precipitation_)
- *μ*_distance_, *μ*_*flow*_, *μ*_precipitation,_ ~ Normal (0,0.3)
- *σ*_distance_, *σ*_*flow*,_ *σ*_*precipitation*_ ~ Exponential (2)
- *κ* ~ Normal (0,50)

We modelled Sorensen indices with a Beta distribution using the mean (μ) and sample size (κ) parameterisation^40^ because similarity indices range between 0 and 1. To make sure location parameter μ is bounded between 0 and 1 we modelled the logit of *μ* in a linear model of the covariates. The terms α_s[comm. 1]_ and α_s[comm. 2]_ are additive varying intercepts that incorporate the dependence resulting from having Sorensen indices calculated with the same sample^39^. By definition, each Sorensen index is paired comparison between two ecological communities, therefore we need to explicitly model the contributions of both communities that comprise each Sorensen index observation. The term *α*_*c*_ is a varying intercept with 11 levels representing each river basin's independent contribution. To capture the influence of the Strahler order difference, we introduced a varying intercept *α*_*o*_ with seven levels representing the independent contribution of Strahler order difference. This approach allows the model to capture expected nonlinear correlations between the logit(*μ*) and Strahler order differences. Flow connection, network distance, and precipitation differences were added as regular covariates to the model. We transformed both covariates to improve model fit and identifiability and to improve run time. Rather than rescaling the covariates by subtracting the mean and dividing by the standard deviation we decided to use values determined by our domain expertise, which improves the interpretability and generalizability of the resulting inferences. We transformed network distance values by subtracting 100 km to observed values and divided the resulting value by 100. We believe that given the study area’s characteristics 100 km is a reasonable threshold beyond which we can expect to find changes in community composition due to ecological drift. Therefore, a slope of, for instance, − 0.10 means that an increase of 1 km in the network distance beyond a baseline of 100 km will decrease Sorensen indices by − 0.10. Precipitation difference values were log-transformed with log(x + 1). We used a log-transformation because the distribution of the values was skewed and presented a second maximum for larger values (Appendix 1—Section X). Afterwards, we subtracted 5.71 (log(300 mm + 1) = 5.71) and divided the resulting value by 5.71. We selected 300 mm because it is the average minimum precipitation value for Mediterranean climates. A slope of − 0.1 indicates that if the precipitation difference increases by 1 mm beyond 300 mm, the Sorensen index will change by − 0.10. The slopes for these three covariates were sampled from a hierarchical distribution (i.e., hyper prior) that generates parameters for all eleven river basins. Slope estimates obtained in this fashion are more precise at the river basin level and usually more robust to extreme observations^41^. We interpreted the posterior distribution of *μ*_distance_, *μ*_*flow*_, and *μ*_precipitation_ as the average effect of the covariate on Sorensen indices if we were to go out into the field to gather vegetation samples from additional river basins.

We used a weakly informative prior following the work by Rodríguez-González et al.^42^. We assumed that a high number of samples would share between 25 and 65% of the species, but also assigned a relatively high probability to lower and higher values. Concerning the covariates, we chose normal distributions for the hyper priors μ_β_ of the slope parameters β with mean zero and a standard deviation of 0.3. These prior choices are also weakly informative allowing for both positive and negative relationships between Sorensen indices and the four covariates. To verify our prior choices we inspected 1000 Sorensen similarity index distributions.

To check that our model captures the data's relevant structure, we compared the observed distribution of the Sorensen indices with the posterior distribution of Sorensen indices. Specifically, we (1) plotted the differences between the posterior distribution and the observed Sorensen indices (i.e., error distributions) conditional on covariates and (2) plotted the posterior distribution of Sorensen indices against the covariates. We checked for systematic deviations that indicated structure in the data that our model was unable to capture. We assessed the importance of the covariates by checking if the corresponding parameter's 95% credibility interval included zero and by evaluating the parameter's magnitude. We chose 95% because it is a standard threshold in both frequentist and Bayesian statistics. However, we do not base our conclusions concerning the importance of the covariate solely on the credibility interval containing zero.

We used the software Stan via the R package “rstan”^43^ and run the models with four independent Markov chains with 1000 warmup iterations and 2000 sampling iterations. To check if our Markov chains were stationary and enabled reasonable posterior expectation value estimators, we performed both qualitative and quantitative diagnostics. In addition, to spot-checking traceplots, we checked that the split potential scale reduction factor (Rhat) was consistent with 1 for all functions of interest and verified that there were no divergent transitions or Markov chains that saturated the maximum tree depth.

Throughout the manuscript, we use the term "retrodictive" instead of "predictive" to refer to the process of comparing predicted results with observed data^44^.

## Results

### Prior predictive checks

**Figure 3:**
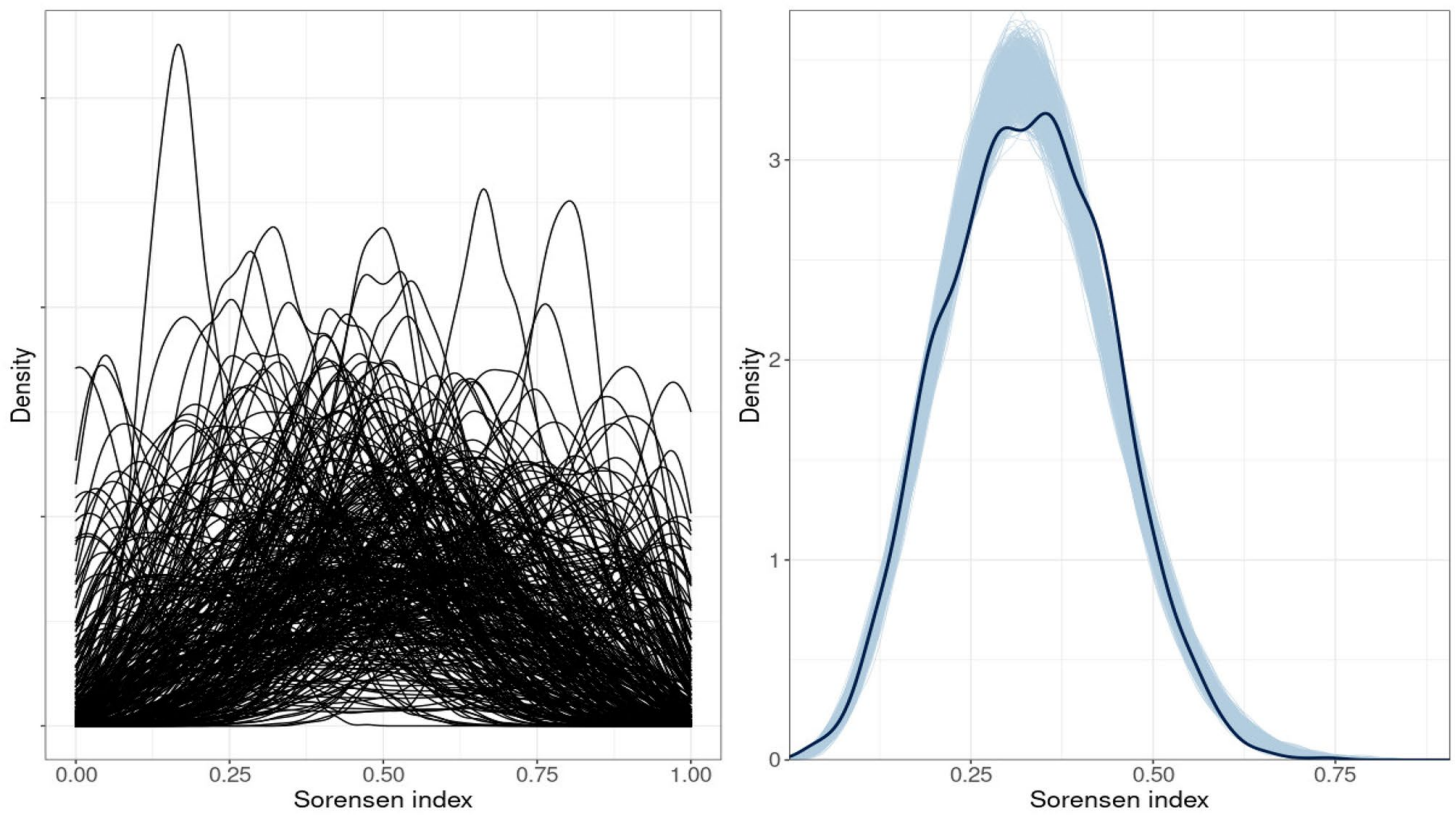
Density plots showing prior predictive distribution (left) and the observed. distribution of Sorensen indices (thick line) against 1000 posterior distributions (thin lines) (right).

We obtained 1000 simulations of Sorensen indices from the prior model and observed that most distributions present a high probability mass between 25 and 65% of the species (Fig. 3 left) which is in accordance with our goal.

### Model validation

The chains were stationary and well mixing with Rhat values of ~ 1. No iterations ended with divergences or saturated the maximum tree depth. The posterior retrodictive distribution of Sorensen indices closely matched the observed distribution of Sorensen indices except for values below 0.05, which are slightly overestimated, and for values above 0.62, which are slightly underestimated. We found no systematic deviations between our data and model (Fig. 3 and Appendix 1).

### Neutral variables

**Figure 4:**
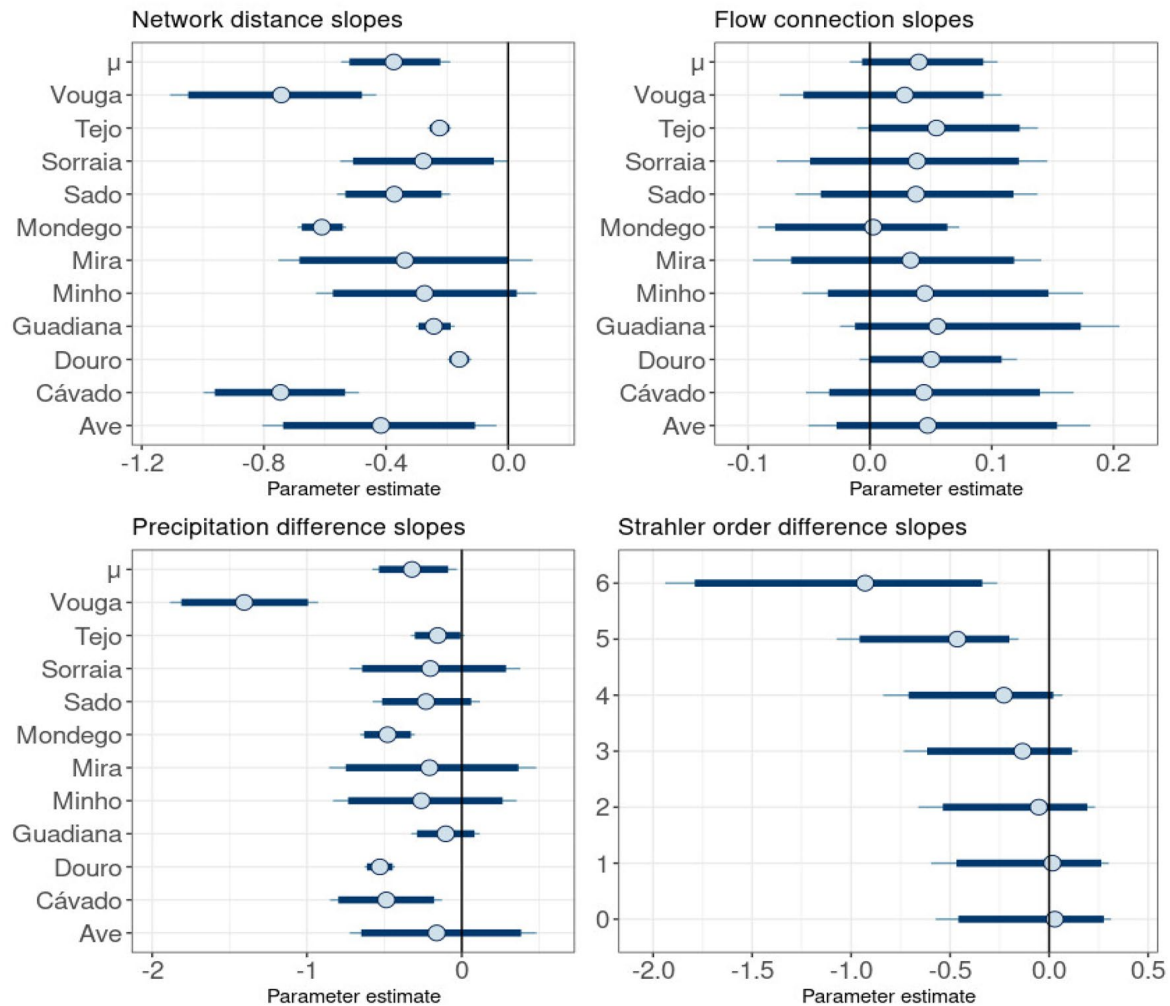
Posterior estimates for the parameters corresponding to the network distance, flow connection, precipitation difference and Strahler order difference. The parameter *μ* is the mean of the normal distribution where slopes are sampled. Dark blue lines represent 95% credibility intervals. The thin light blue line represents the complete distribution of the parameters. The dot represents the marginal posterior mean.

Network distance was negatively associated with Sorensen similarity indices, with mean slope estimates ranging between − 0.16 for the Douro and − 0.75 for the Cávado and Vouga basins. In Mira and Minho basins, a small part of the 95% credibility intervals crosses zero, which means there is a small probability that the slopes are zero or slightly positive (Fig. 4). The estimate for *μ*_distance_ was − 0.37 with a 95% credibility interval [− 0.55, − 0.19]. We can interpret this results as follows: beyond a threshold distance of 100 km, if the network distance increases by 1 km, the Sorensen index decreases by 37%.

Vegetation samples connected by streamflow seem to be slightly more similar than those that are not. Mean slope estimates were relatively small but predominantly positive except for Mondego basin's slope, whose estimate was very close to zero. For the remaining basins, mean estimates ranged between 0.02 and 0.06 (Fig. 4). All 95% credibility intervals crossed 0, which means that weakly negative contributions are also consistent with the data. The mean estimate for μ_flow_ is 0.04 [− 0.02, 0.10], which suggests that when all covariates remain constant the Sorensen index increases by 4% when two vegetation samples are connected. However, decreases of 2% or increases of 10% are also consistent with the data, but less likely.

### Niche-based variables

Strahler order difference had a negative association with community similarity. Sorensen indices seem to decrease linearly when Strahler order differences change between 0 and 4, but this decrease is steeper when Strahler order increases to 5 and mainly to 6 (Fig. 4). For instance, when two vegetation samples have a Strahler order difference of 6, Sorensen indices will on average be 98% smaller.

Higher differences in precipitation are associated with lower levels of community similarity. The mean estimates for precipitation difference slopes were negative, with mean values ranging between − 0.53 and 0.12 (Fig. 4). However, in six out of eleven basins, credibility intervals crossed zero, indicating the effect could also be weakly positive with varying degrees of probability. For instance, over 30% of Mira's basin parameter distribution is on the right side of zero. The estimate for *μ*_precipitation_ was − 0.32 [− 0.55, − 0.19] which means that when precipitation difference increases by 1 mm beyond a threshold of 300 mm, Sorensen indices decrease on average by 32%.

A slope of − 0.1 indicates that if the precipitation difference increases by 1 mm beyond 300 mm (log(300 mm + 1) = 5.71), the Sorensen index will change by − 0.10.

## Discussion

In this work, we analysed the influence of neutral and niche-based factors on the distance decay of community similarity (DDCS) in riparian plant communities of eleven river basins in continental Portugal. We considered two neutral covariates, network distance and flow connection and two niche-based covariates, precipitation difference, and Strahler order difference.

Network distance was positively associated with lower community similarity. The ecological neutral theory argues the DDCS is caused by ecological drift, which consists of random extinctions, species replacement^12,45^, and by random dispersal coupled with dispersal limitations^46^. As distances increase, the probability of successful seed dispersal decreases^47–49^, which can explain why community similarity decreases with distance. Still, we cannot rule out the possibility that an unmeasured covariate correlated with network distance may have caused this result. For instance, drought-related habitat fragmentation could hinder seed dispersal^50^ and cause identical reductions in community similarity. However, we believe that the covariate precipitation difference already captures drought-related effects.

Sorensen indices from vegetation samples that are connected by water are 4% higher than those that are not. Indeed, flow connectivity is responsible for high level of diversity in fluvial ecosystems^51^. Most riparian plant species have the ability to disperse seeds through water^52^, explaining why communities connected by streamflow share a higher number of species, and the pattern of increased richness in a downstream direction^53–55^. This result may be also caused by an association between streamflow and katabatic winds. Air masses that are thermally forced from higher to lower altitudes usually travel along river networks^56^. These air masses can transport seeds and pollinators along with river networks, thus increasing community similarity^57,58^. Another possibility is that pollinators may be more prone to move along river corridors following their foraging preferences^59^, thus contributing to higher community similarities.

Community similarity was higher between vegetation samples experiencing lower differences in precipitation. Continental Portugal exhibits a high precipitation gradient. This spatial and temporal variability in precipitation results in some regions/periods of the year experiencing frequent droughts during the summer and others experiencing intense floods during the winter^20^. Plant communities have thus adapted to these conditions. In drier areas, riparian ecosystems harbor a higher number of terrestrial plants and proportionally fewer strictly riparian or aquatic species^60^. In wetter regions, the prevalence of species adapted to waterlogged and frequently flooded is considerably higher^60^. This community composition pattern may cause a negative relationship between community similarity and precipitation difference.

Overall, community similarity decreases with increasing Strahler order differences. This result suggests that riparian plant communities located closer to the river's source (lower Strahler order) tend to differ from those closest to the river's mouth (higher Strahler order), even after accounting for network distance. Moreover, this difference increases with increasing separation in river networks, particularly when Strahler order differences increase to five and six. Differences in community composition may be explained by differences in environmental features upstream and downstream^61^ such as elevation, channel gradient, valley constraints, geomorphic processes, and substrate diversity^20,21,38^. An alternative explanation for this result are confluence effects^62^. When two rivers meet, water and sediment influx changes affect channel and floodplain morphology and alter the composition of riparian plant communities, which could produce similar results to those we observed.

In summary, our model results suggest that community similarity changes are associated with both environmental and neutral factors. Both niche-based and neutral variables were associated with non-null changes in Sorensen indices. Network distances and Strahler order differences had the largest effect sizes, followed by precipitation difference and flow connection. Overall, the results seem to be consistent with the continuum hypothesis that states that niche and neutral factors are at opposite ends of a continuum^14^. The results from this study contribute to improving our knowledge of the processes that shape riparian ecosystems and underline the importance of considering both environmental and neutral factors when analysing changes in community composition.

## Supplementary Information

Supplementary Information.
