# Supplementary material for: Analysing the distance decay of community similarity in river networks using Bayesian methods
Source: Sci Rep. 2021 Nov 4;11:21660. doi: 10.1038/s41598-021-01149-x (PMC8569194; doi:10.1038/s41598-021-01149-x)
Supplement: Supplementary file 1 — Supplementary Information. [file 41598_2021_1149_MOESM1_ESM.pdf]

Appendix 1 - Distance decay of community similarity in river networks: a Bayesian approach

Filipe S. Dias (CIBIO-InBIO) Michael Betancourt (Symplectomorphic, LLC)
Patricia Rodríguez-González (CEF/ISA) Luís Borda de Água (CIBIO-InBIO)

Contents

1. Introduction . . . 1
2. Preamble . . . 1
3. Dataset . . . 2
4. Exploratory data analyis . . . 2
    4.1 Inspect the distribution of Sorenson indices and covariates . . . 2
    4.2 Plot Sorensen indices against covariates . . . 4
5. Modeling . . . 8
    5.1 Data preparation . . . 8
    5.2 The model . . . 9
    5.3 Prior Predictive checks . . . 11
    5.4 Fit the model . . . 12
    5.5 Model validation . . . 12
        5.5.1 Run diagnostics . . . 12
        5.5.2 Posterior retrodictive checks . . . 13
            5.5.2.1 Observed against predicted Sorenson indices . . . 13
            5.5.2.2 Residuals and predicted Sorensen indices against network distance . . . 15
            5.5.2.3 Residuals and predicted Sorensen indices against flow connection . . . 15
            5.5.2.3 Residuals and predicted Sorensen indices against precipitation difference 16
            5.5.2.4 Residuals and predicted Sorensen indices against Strahler order difference 17
            5.5.2.5 Residuals and predicted Sorenson indices against basin intercepts . . . 18
            5.5.2.6 Residuals and predicted Sorensen indices against sample intercepts . . . 18
    5.6 Parameter estimates . . . 19
        5.6.1 Network distance slopes . . . 20
        5.6.2 Flow connection . . . 21
        5.6.3 Precipitation difference . . . 21
        5.6.4 Strahler order difference . . . 22

1. Introduction

In this document we include the R code for reproducing the analysis presented in "Distance decay of community similarity in river networks: a Bayesian approach". Make sure to download "data.csv", "model.stan" and "stan_utility.R".

2. Preamble

We start by loading all required packages.

```r
library(knitr)
library(rstan)
library(bayesplot)
library(ggplot2)
library(dplyr)
library(gridExtra)
library(boot)
```

3. Dataset

Then, we load the dataset.

```r
dataset_annex <- read.csv("dataset_annex.csv")
```

1. `sor` - Sorenson-Dice similarity index
2. `net_dist_km` - network distance between a pair of of vegetation censuses (km)
3. `euc_dist_km` - euclidean distance between vegetation censuses (km)
4. `flow_conn` - 1-samples are flow connected, 0 - samples are not flow connected
5. `pp_diff` - difference in natural precipitation between the pair of vegetation samples (mm/year)
6. `strahler_diff` - Strahler order difference between vegetation samples
7. `sample_idx1` - snique identifier for the first vegetation sample
8. `sample_idx2` - unique identifier for the second vegetation sample
9. `basin_name` - name of the river basins 10.`basin_id` - same as `basin_name` but in integer form

4. Exploratory data analyis

4.1 Inspect the distribution of Sorenson indices and covariates

```r
grid.arrange(
  ggplot(data=dataset_annex,mapping=aes(x=sor))+geom_histogram()+
  labs(title="Histogram of observed Sorenson indices",x="Sorenson index")+
  theme_bw(),

  ggplot(data=dataset_annex,mapping=aes(x=net_dist_km))+geom_histogram()+
  labs(title="Histogram of observed network distances",x="network distance (km)")+
  theme_bw(),

  ggplot(data=dataset_annex,mapping=aes(as.factor(flow_conn)))+geom_bar()+
  labs(title="Frequency of flow connection categories",x="0 - not connected, 1 - connected")+theme_bw()

  ggplot(data=dataset_annex,mapping=aes(x=pp_diff))+geom_histogram()+
  labs(title="Histogram of observed Precipitation differences",
  x="precipitation difference(mm/year)")+
  theme_bw(),

  ggplot(data=dataset_annex,mapping=aes(strahler_diff))+geom_bar()+
  labs(title="Frequency of Strahler order difference",x="Strahler order difference")+
  theme_bw(),
  ncol=2)
```

```
## `stat_bin()` using `bins = 30`. Pick better value with `binwidth`.
## `stat_bin()` using `bins = 30`. Pick better value with `binwidth`.
## `stat_bin()` using `bins = 30`. Pick better value with `binwidth`.
```

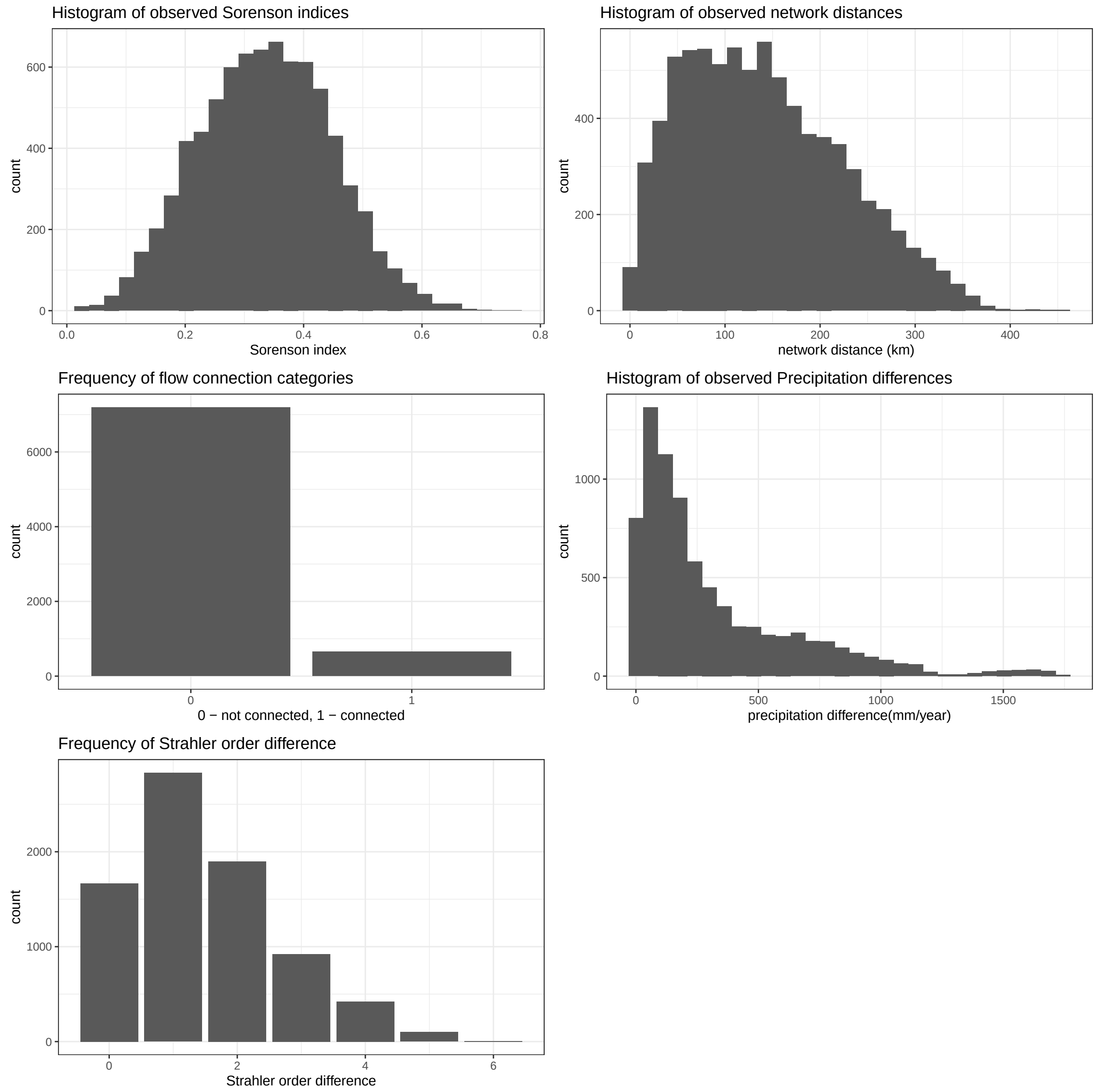

The histogram with observed Precipitation differences shows what appears to be a second maximum in larger values, so we decided to apply a log(x+1) transformation.

```
ggplot(data=dataset_annex,mapping=aes(x=log(pp_diff+1)))+geom_histogram()+
  labs(title="Histogram of observed Precipitation differences (logx+1 transformed)",
       x="precipitation difference(mm/year)")+theme_bw()
```

```
## `stat_bin()` using `bins = 30`. Pick better value with `binwidth`.
```

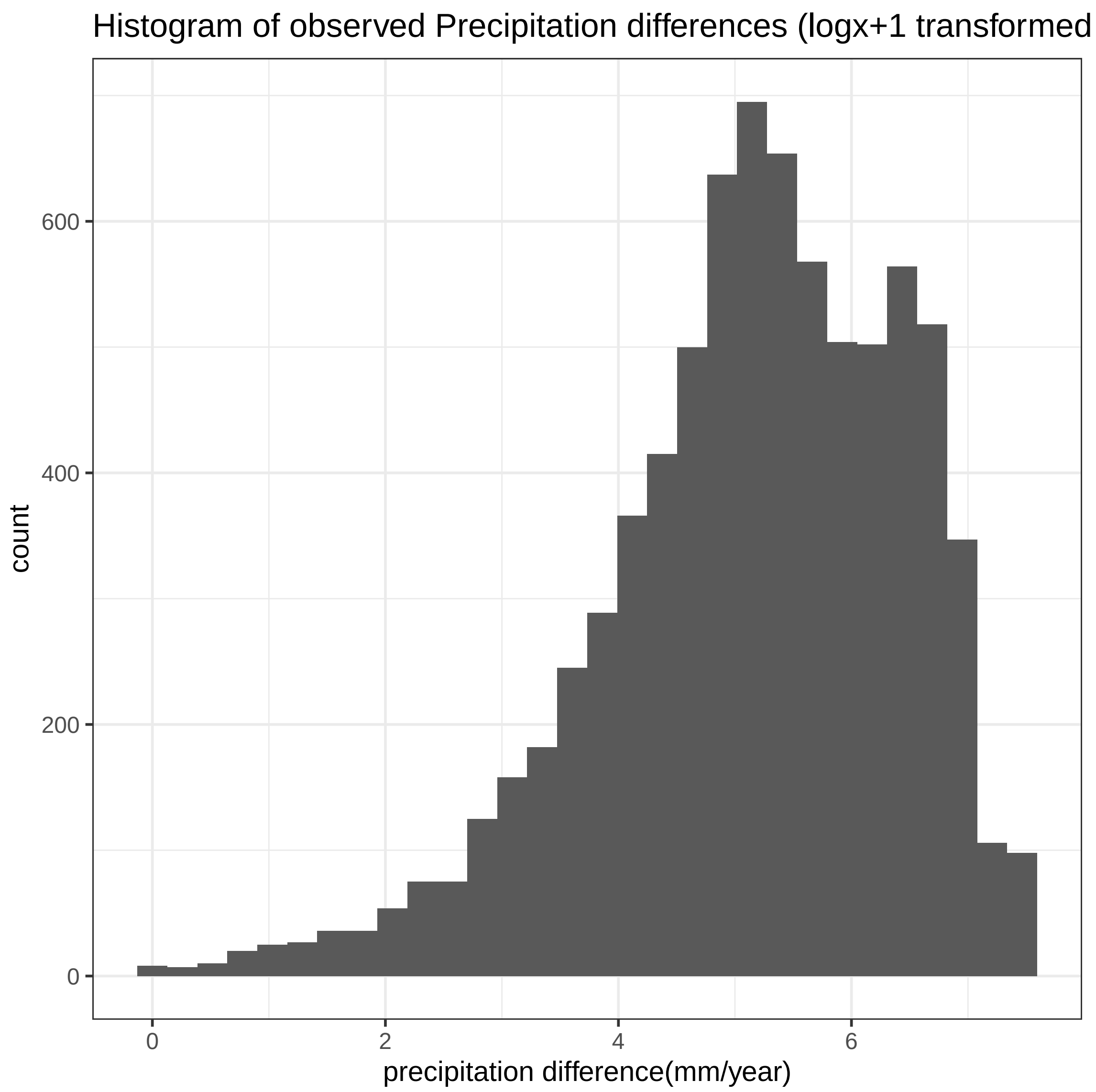

4.2 Plot Sorensen indices against covariates

```
ggplot(data=dataset_annex,mapping=aes(y=sor,x=net_dist_km))+
  geom_point(color="red",alpha = 0.10)+facet_wrap(vars(basin_name))+
  labs(x="Network distance (km)",y="Sorenson index")+theme_bw()
```

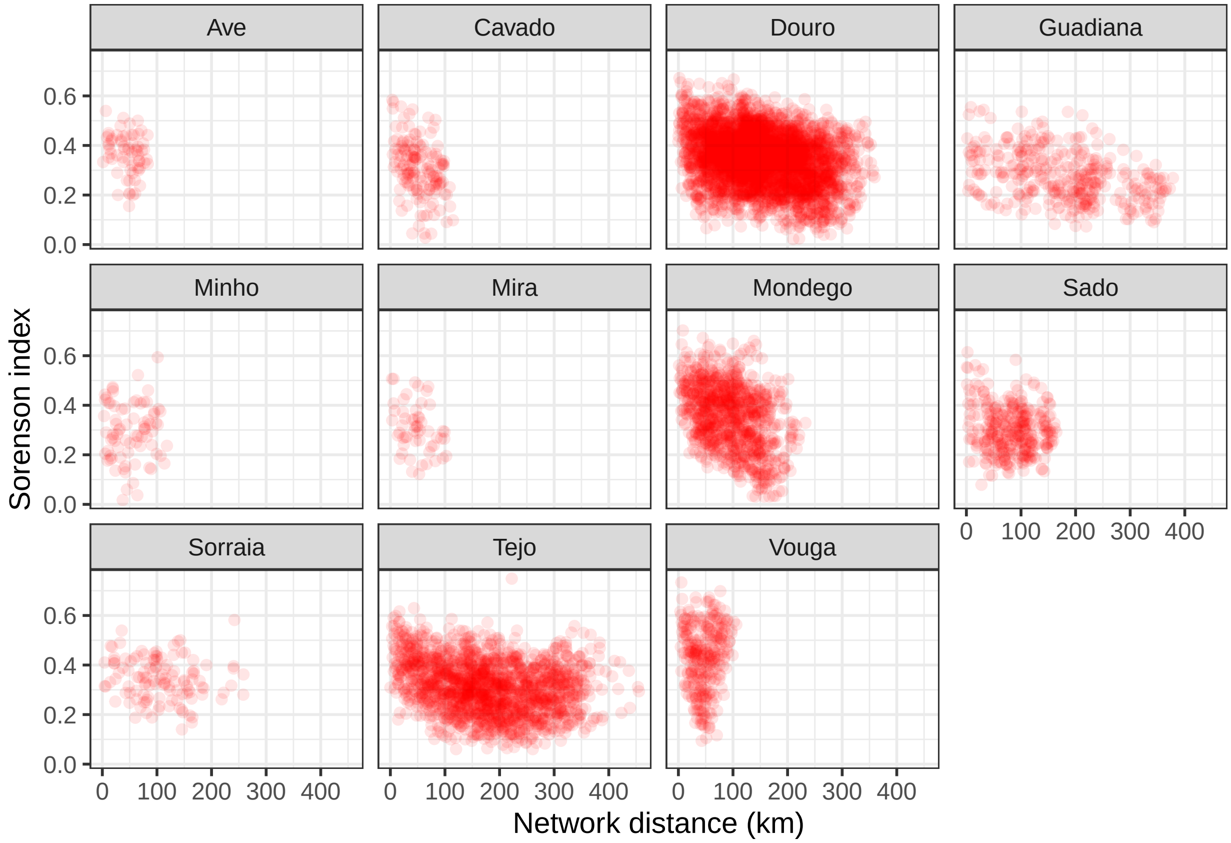


```
ggplot(data=dataset_annex,mapping=aes(y=sor,x=factor(flow_conn)))+
geom_boxplot()+facet_wrap(vars(basin_name))+
labs(y="Sorenson index",x="Flow connection (1-Yes, 0 - No)")+
  theme_bw()
```

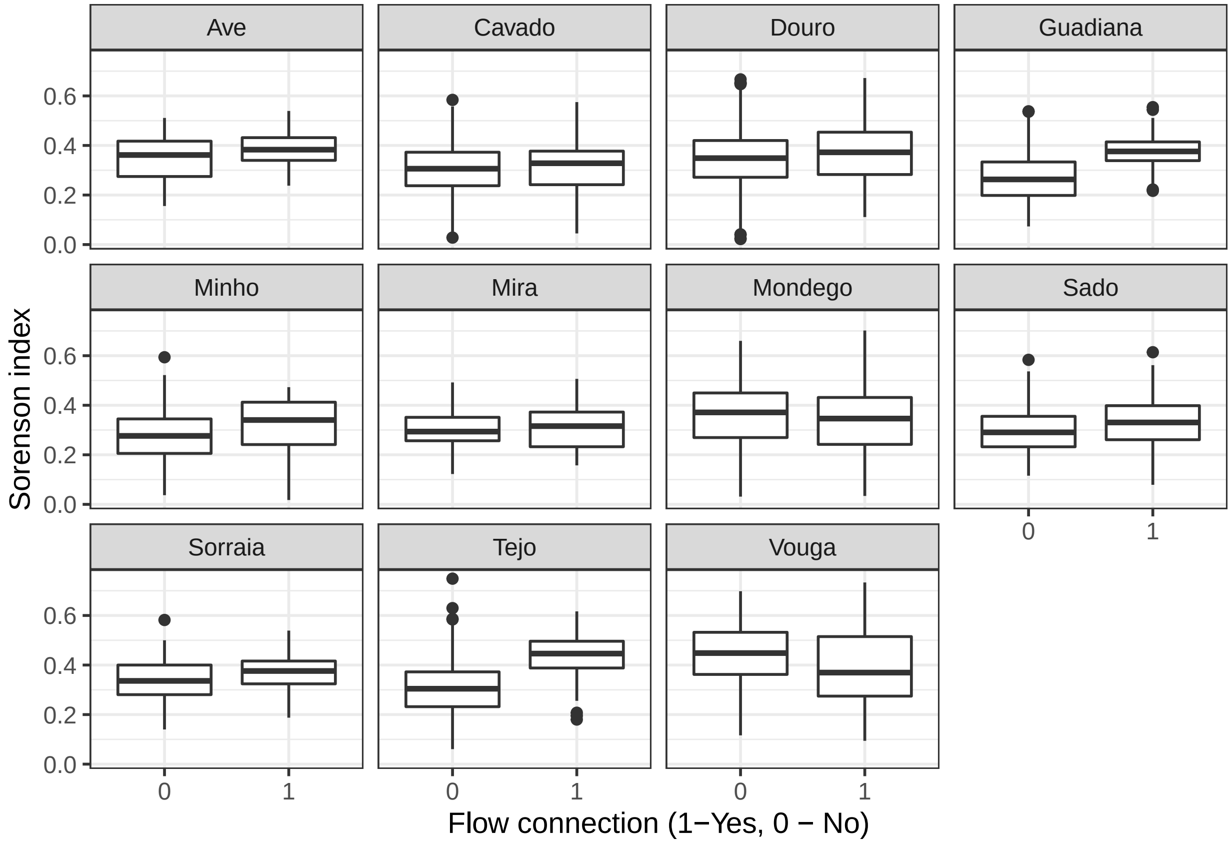


```
ggplot(data=dataset_annex,mapping=aes(y=sor,x=log(pp_diff+1)))+
  geom_point(color="red",alpha = 0.10)+facet_wrap(vars(basin_name))+
  labs(x="Precipitation difference (mm/year)",y="Sorenson index")+theme_bw()
```

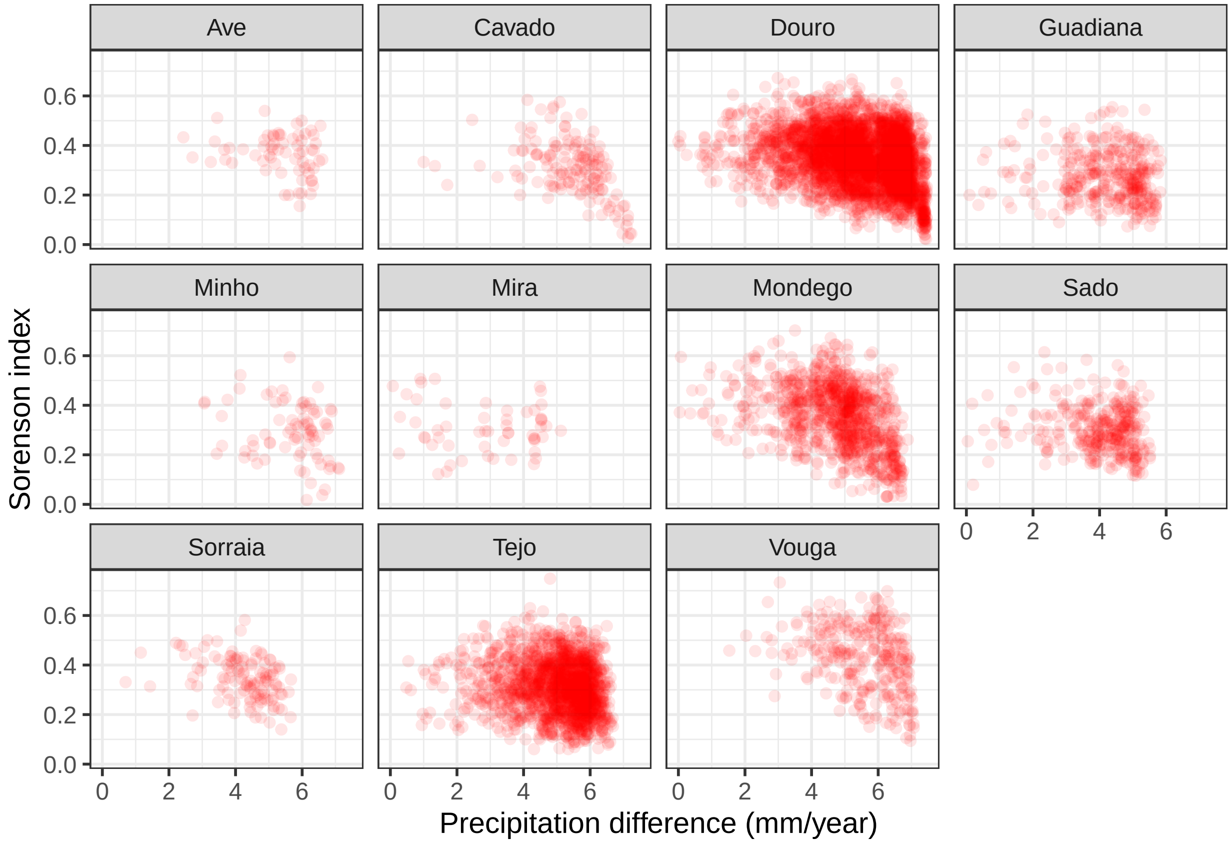


```
ggplot(data=dataset_annex,mapping=aes(y=sor,x=strahler_diff))+
geom_point(color="red",alpha = 0.10)+facet_wrap(vars(basin_name))+
labs(x="Strahler order difference",y="Sorenson index")+theme_bw()
```

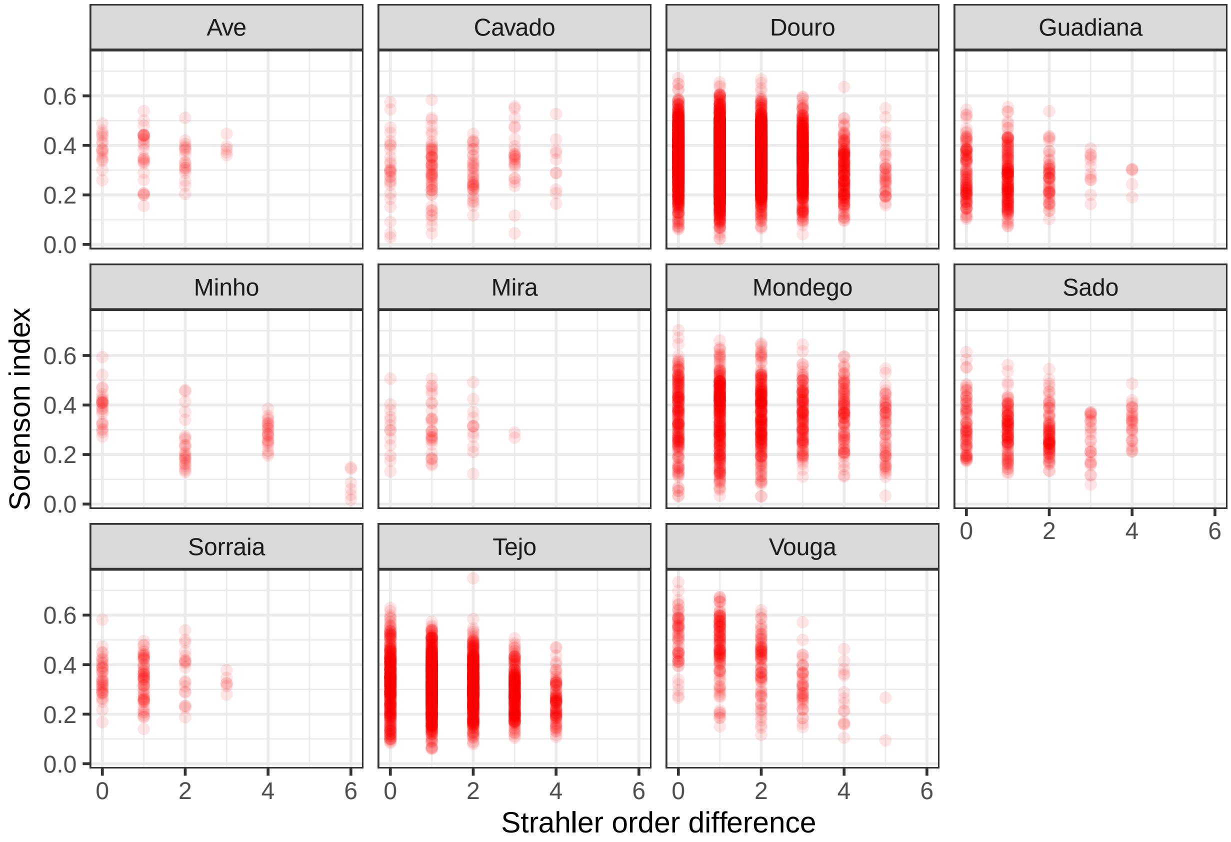

5. Modeling

5.1 Data preparation

We need to filter observations for which the ratio between the network distance and the euclidean distance is <= 2.

```
dataset_annex$frac<-dataset_annex$net_dist_km / dataset_annex$euc_dist_km
df<-filter(dataset_annex,frac<=2)
```

Then we add the log transformed precipitation difference

```
df$logpp_diff<-log(df$pp_diff+1)
```

Now, we create an input list for running the model.

```
data_list<-list(N_obs = length(df$sor),
           sor = df$sor,
           net_dist_km = (df$net_dist_km-100)/100,
           N_flow = 2,
           flow_conn = df$flow_conn + 1,
           N_strahlers = max(df$strahler_diff + 1),
           strahler_diff = df$strahler_diff + 1,
           pp_diff = (df$logpp_diff-5.71)/5.71,
           N_basins = max(df$basin_id),
           basin_idx = df$basin_id,
```

```
            N_samples = max(df$sample_id1),
            sample_idx1 = df$sample_id1,
            sample_idx2 = df$sample_id2
)
```

5.2 The model

```
data {
int<lower=1> N_obs;
vector[N_obs] sor;
vector[N_obs] net_dist_km;
vector[N_obs] pp_diff;
int<lower=1> N_flow;
vector[N_obs] flow_conn;
int<lower=1> N_strahlers;
int<lower=1, upper=N_strahlers> strahler_diff[N_obs];
int N_basins;
int<lower=1, upper=N_basins> basin_idx[N_obs];
int N_samples;
int<lower=1, upper=N_samples> sample_idx1[N_obs];
int<lower=1, upper=N_samples> sample_idx2[N_obs];
}

parameters {

real a_baseline;
vector[N_basins] a_basin;
real<lower=0> sigma_basin;

vector[N_samples] a_sample;
real<lower=0> sigma_sample;

vector[N_strahlers] a_strahler_ncp;
real<lower=0> sigma_strahler;

vector[N_basins] bflow_ncp;
real mu_bflow;
real<lower=0> sigma_bflow;

vector[N_basins] bnet;
real mu_bnet;
real<lower=0> sigma_bnet;

vector[N_basins] bpp;
real mu_bpp;
real<lower=0> sigma_bpp;

real<lower=0> kappa;
}

transformed parameters {

vector[N_strahlers] a_strahler = sigma_strahler * a_strahler_ncp;
```

```
vector[N_basins] bflow = mu_bflow+sigma_bflow*bflow_ncp;

vector[N_obs] mu;

mu = a_baseline
+ a_sample[sample_idx1] + a_sample[sample_idx2]
+ a_basin[basin_idx]
+ a_strahler[strahler_diff]
+ bflow[basin_idx].* flow_conn
+ bnet[basin_idx] .* net_dist_km
+ bpp[basin_idx] .* pp_diff;
}

model {

a_baseline ~ normal(0, 0.3);

a_sample ~ normal(0, sigma_sample);
sigma_sample ~ exponential(4);

a_basin ~ normal(0, sigma_basin);
sigma_basin ~ exponential(4);

a_strahler_ncp ~ normal(0, 1);
sigma_strahler ~ exponential(4);

bnet~normal( mu_bnet, sigma_bnet );
mu_bnet~normal(0,0.3);
sigma_bnet ~ exponential(4);

bflow_ncp~normal(0,1);
mu_bflow~normal(0,0.3);
sigma_bflow ~ exponential(4);

bpp~normal(mu_bpp,sigma_bpp);
mu_bpp~normal(0,0.3);
sigma_bpp ~ exponential(4);

kappa ~ normal(0, 50);
sor ~ beta_proportion(inv_logit(mu), kappa);
}

generated quantities{

real sor_pred[N_obs];
real res[N_obs];

for(i in 1:N_obs){
sor_pred[i]= beta_proportion_rng(inv_logit(mu[i]), kappa);
}

for(i in 1:N_obs){
res[i]= sor_pred[i]-sor[i];
```

```
}
}
```

5.3 Prior Predictive checks

The prior model assumes that most Sorensen indices will be around 0.25 - 0.75. To check this we plotted 300 prior predictive distributions:

```
sample_prior<-function(){

  sigma_basin = rexp(1,4)
  sigma_sample = rexp(1,4)
  sigma_strahler = rexp(1,4)
  sigma_basin_strahler = rexp(1,4)



  a_baseline = rnorm(1,0,0.3)
  a_basin = rnorm(11,0, sigma_basin)
  a_sample = rnorm(403,0, sigma_sample) #403 instead of 338 because sample_idx are not sequential

  a_strahler=rnorm(7,0,sigma_strahler)

  a_basin_strahler=rnorm(54,0,sigma_basin_strahler)

  mu_bnet=rnorm(1,0,0.3)
  sigma_bnet_basin=rexp(11,4)

  mu_bpp=rnorm(1,0,0.3)
  sigma_bpp_basin=rexp(1,4)

  mu_bflow=rnorm(1,0,0.3)
  sigma_bflow_basin=rexp(1,4)

  bnet = rnorm(11,mu_bnet,sigma_bnet_basin)
  bpp = rnorm(11,mu_bpp,sigma_bpp_basin)
  bflow = rnorm(11,mu_bflow,sigma_bflow_basin)


  kappa = abs(rnorm(1,0,50))
  kappa<-rep(kappa, 3657) #3657 is the total number of observations

  mu =  inv.logit(a_baseline + a_sample[data_list$sample_idx1] +
                    a_sample[data_list$sample_idx2]+
                    a_basin[data_list$basin_idx]+
                    a_strahler[data_list$strahler_diff]+
                    a_basin_strahler[data_list$strahler_basin_idx]+
                    bnet[data_list$basin_idx] * data_list$net_dist_km +
                    bpp[data_list$basin_idx] * data_list$pp_diff+
                    bflow[data_list$basin_idx]*data_list$flow_conn)

  alfa=mu*kappa
  beta = (1-mu)*kappa
```

```
  pred<-rbeta(3657, alfa, beta)
  return(pred)
}
```

```
samples<-replicate(300, sample_prior(), simplify=FALSE)
p<-ggplot()
for(i in 1:300){
  input<-samples[[i]]
  input<-data.frame(pred=input)
  p<-p+geom_density(input,mapping=aes(pred))
}
p<-p+labs(x="Sorensen index",y="Density")+theme_bw()
p+theme(axis.text.y=element_blank())
```

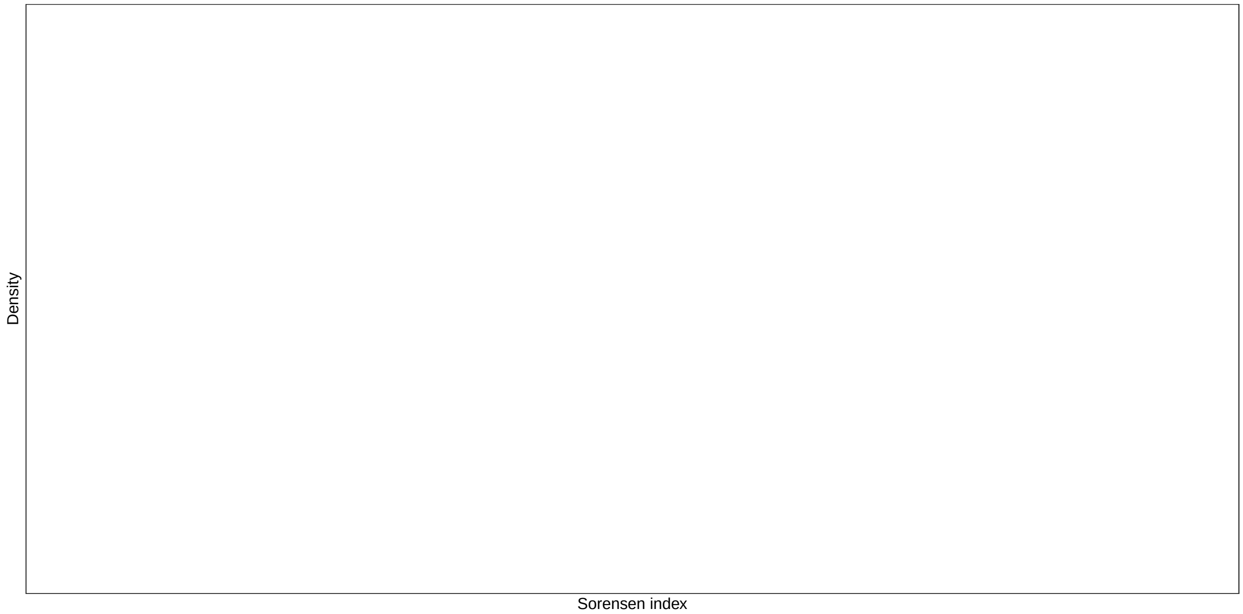

Most prior distributions have high probability mass around 0.25-0.75, and few distributions assign high probability to values close to 0 or 1.

5.4 Fit the model

```
library(rstan)
rstan_options(auto_write = TRUE)
options(mc.cores = parallel::detectCores())
parallel:::setDefaultClusterOptions(setup_strategy = "sequential")
fit <- stan(file='model.stan', data=data_list)
```

5.5 Model validation

5.5.1 Run diagnostics We validated the model by checking 1) transitions that ended with a divergence, 2) transitions that ended prematurely due to maximum tree depth limit, 3) the energy Bayesian fraction of missing information (E-BFMI), 4) the effective sample size per iteration and 5) the potential scale reduction factors.

```
util <- new.env()
source('stan_utility.R', local=util)
util$check_all_diagnostics(fit)
```

```
## [1] "n_eff / iter looks reasonable for all parameters"
## [1] "Rhat looks reasonable for all parameters"
## [1] "0 of 4000 iterations ended with a divergence (0%)"
## [1] "31 of 4000 iterations saturated the maximum tree depth of 10 (0.775%)"
## [1] "  Run again with max_depth set to a larger value to avoid saturation"
## [1] "E-FMI indicated no pathological behavior"
```

5.5.2 Posterior retrodictive checks We plotted predicted Sorensen indices and residuals against observed Sorensen indices and covariates, and looked for systematic deviations in the plots. We start by preparing the required data for running the posterior retrodictive checks.

```
sor_pred<-as.matrix(fit,"sor_pred")
residuals<-as.matrix(fit,"res")
sor_mean_pred<-apply(sor_pred,2,mean)
res_mean<-apply(residuals,2,mean)
sor<-data_list$sor
pp_diff<-data_list$pp_diff
net_dist_km<-data_list$net_dist_km
strahler_diff<-data_list$strahler_diff
flow_conn<-data_list$flow_conn
basin_idx<-data_list$basin_idx
sample_idx1<-data_list$sample_idx1
sample_idx2<-data_list$sample_idx2
```

5.5.2.1 Observed against predicted Sorenson indices Next, we make density plots showing the observed distribution of Sorenson indices (thicker line) against 1000 posterior distributions.

```
ppc_dens_overlay(sor,sor_pred[1:1000,])+labs(x="Sorensen index",y="Density")+
  theme_bw()+
  theme(axis.text=element_text(size=14),axis.title=element_text(size=16))+
  legend_none()
```

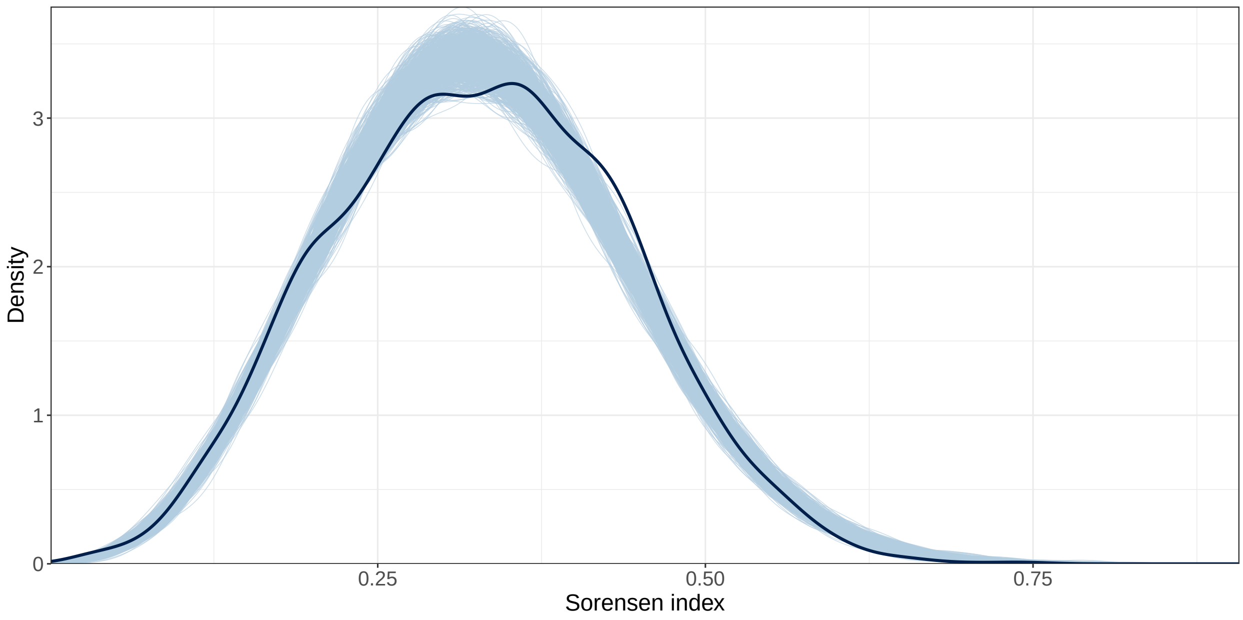

Then, we plot 1) residuals (dark blue points) and its distribution (light blue lines) against observed Sorensen indices, and 2) Predicted Sorenson indices (dark blue points) and its distribution (light blue lines) against observed Sorensen indices.

```
grid.arrange(
ppc_intervals(res_mean,residuals,sor)+labs(y="Residuals (Predicted - Observed)",
                                           x="Observed Sorenson indices")+
                                            theme_bw()+hline_0()+legend_none(),
ppc_intervals(sor_mean_pred,sor_pred,sor)+labs(y="Predicted Sorenson indices",
                                           x="Observed Sorenson indices")+
                                        theme_bw()+ abline_01()+legend_none(),
ncol=2)
```

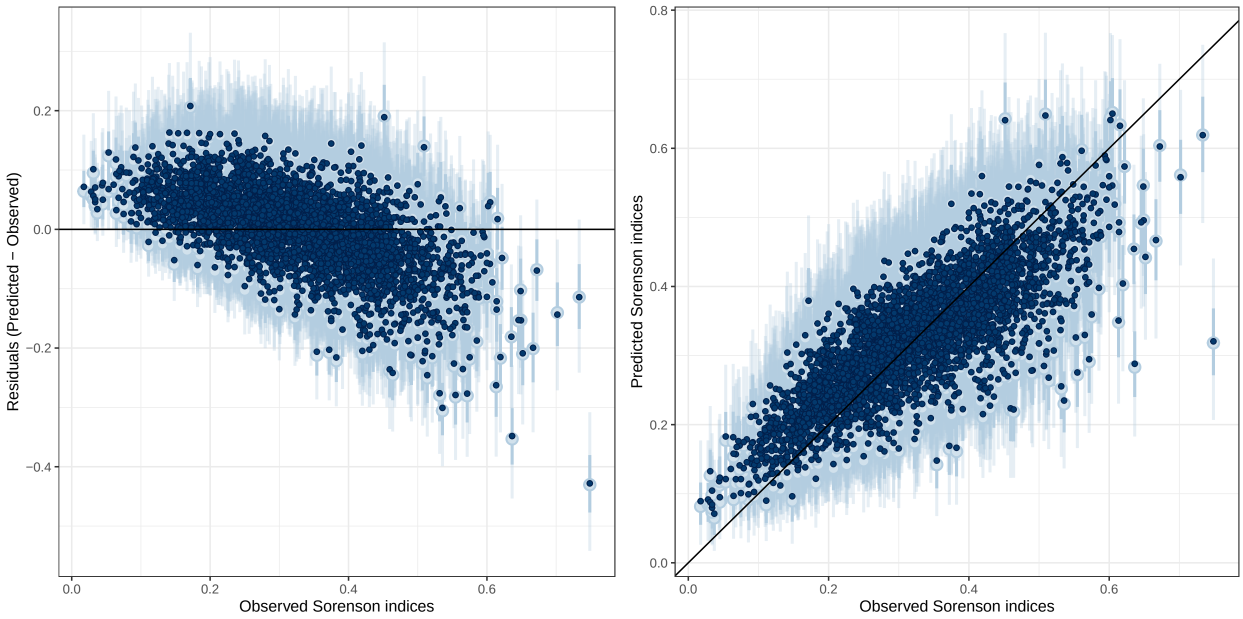

Based on visual inspection we concluded that there is a good match between observed Sorenson indices and

predicted Sorenson indices. However, values below 0.05 are a bit overestimated and values above 0.62 which are slightly underestimated.

5.5.2.2 Residuals and predicted Sorensen indices against network distance This plot shows 1) Residuals (dark blue points) and its distribution (light blue lines) plotted against network distance, and 2) Predicted Sorenson indices (dark blue points) and its distribution (light blue lines) against network distance.

```
grid.arrange(
  ppc_intervals(res_mean,residuals,net_dist_km)+labs(y="Residuals (Predicted - Observed)",
                                                     x="Network distance")+
                                                      theme_bw()+hline_0()+legend_none(),
  ppc_intervals(sor_mean_pred,sor_pred,net_dist_km)+labs(y="Predicted Sorenson indices",
                                                     x="Network distance")+
                                                                   theme_bw()+legend_none(),
  ncol=2)
```

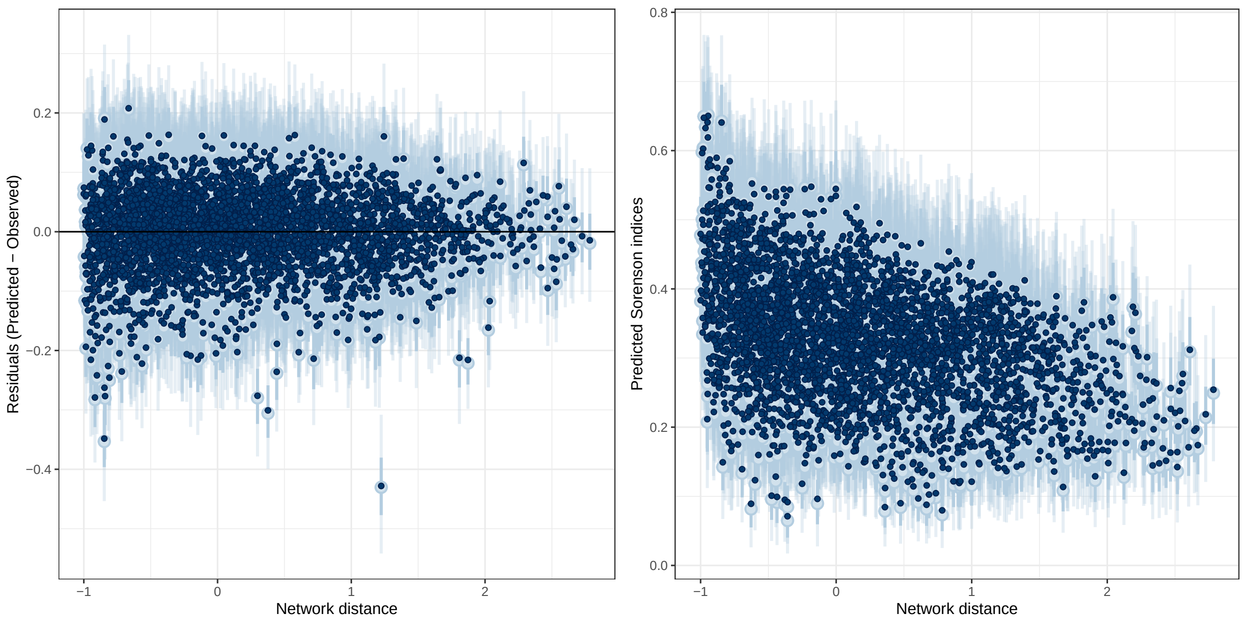

We see no systematic deviations.

5.5.2.3 Residuals and predicted Sorensen indices against flow connection This plot shows 1) Residuals (dark blue points) and its distribution (light blue lines) plotted against flow connection, and 2) Predicted Sorensen indices (dark blue points) and its distribution (light blue lines) against flow connection.

```
grid.arrange(
  ppc_intervals(res_mean,residuals,flow_conn)+labs(y="Residuals (Predicted - Observed)",
                                                     x="Flow connection")+
                                                      theme_bw()+hline_0()+legend_none(),
  ppc_intervals(sor_mean_pred,sor_pred,flow_conn)+labs(y="Predicted Sorenson indices",
                                                     x="Flow connection")+
                                                      theme_bw()+legend_none(),
  ncol=2)
```

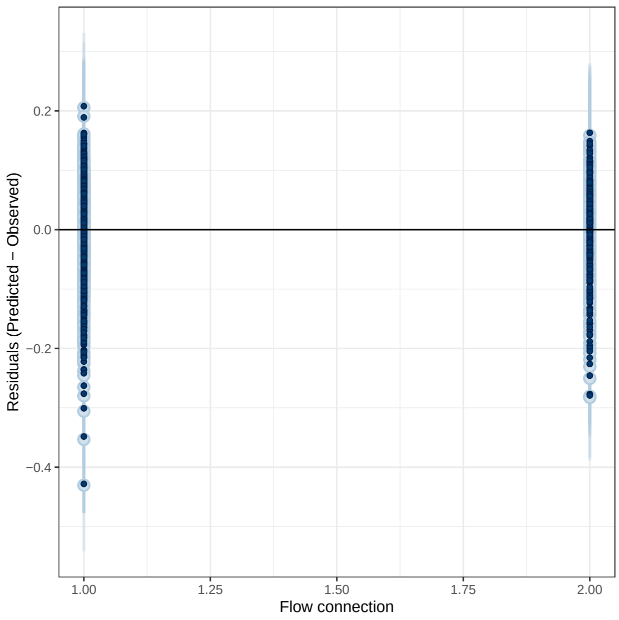

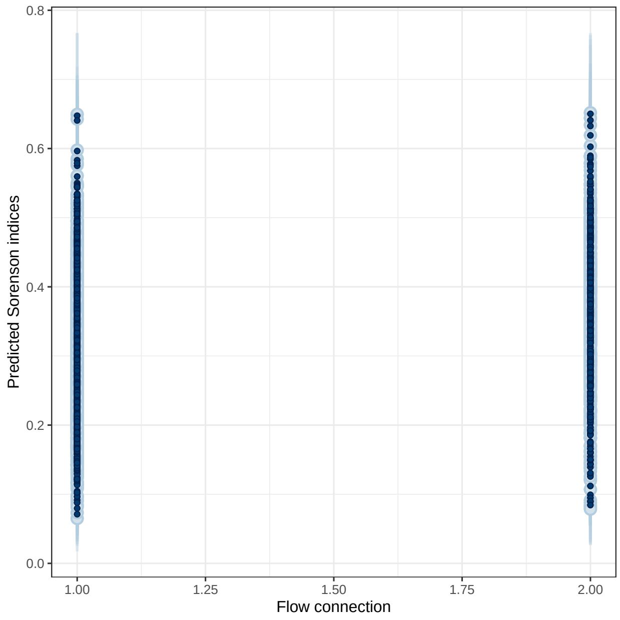

We see no systematic deviations.

5.5.2.3 Residuals and predicted Sorensen indices against precipitation difference This plot shows 1) Residuals (dark blue points) and its distribution (light blue lines) plotted against precipitation difference, and 2) Predicted Sorensen indices (dark blue points) and its distribution (light blue lines) against precipitation difference.

```
grid.arrange(
  ppc_intervals(res_mean,residuals,pp_diff)+labs(y="Residuals (Predicted - Observed)",
                                                 x="Precipitation difference")+
  theme_bw()+hline_0()+legend_none(),
  ppc_intervals(sor_mean_pred,sor_pred,pp_diff)+labs(y="Predicted Sorensen indices",
                                                 x="Precipitation difference")+
 theme_bw()+legend_none(),
ncol=2)
```

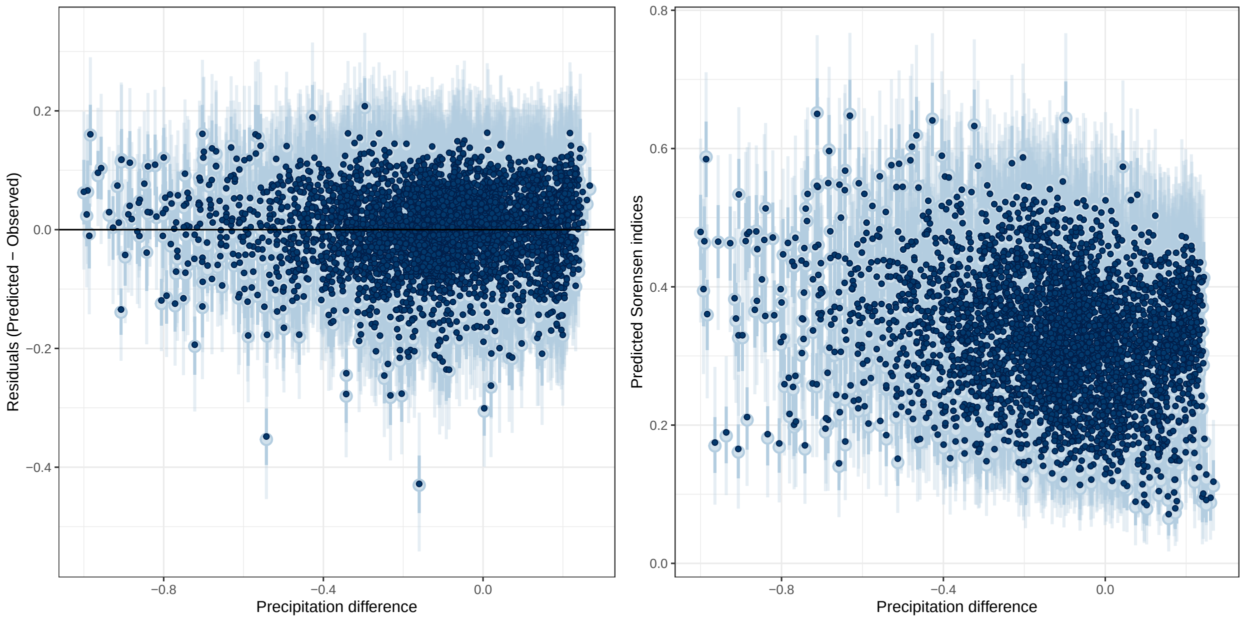

We see no systematic deviations.

```
grid.arrange(
  ppc_intervals(res_mean,residuals,strahler_diff)+labs(y="Residuals (Predicted - Observed)",
                                                        x="Precipitation difference")+
  theme_bw()+hline_0()+legend_none(),
  ppc_intervals(sor_mean_pred,sor_pred,strahler_diff)+labs(y="Predicted Sorensen indices",
                                                        x="Precipitation difference")+
 theme_bw()+legend_none(),
ncol=2)
```

5.5.2.4 Residuals and predicted Sorensen indices against Strahler order difference

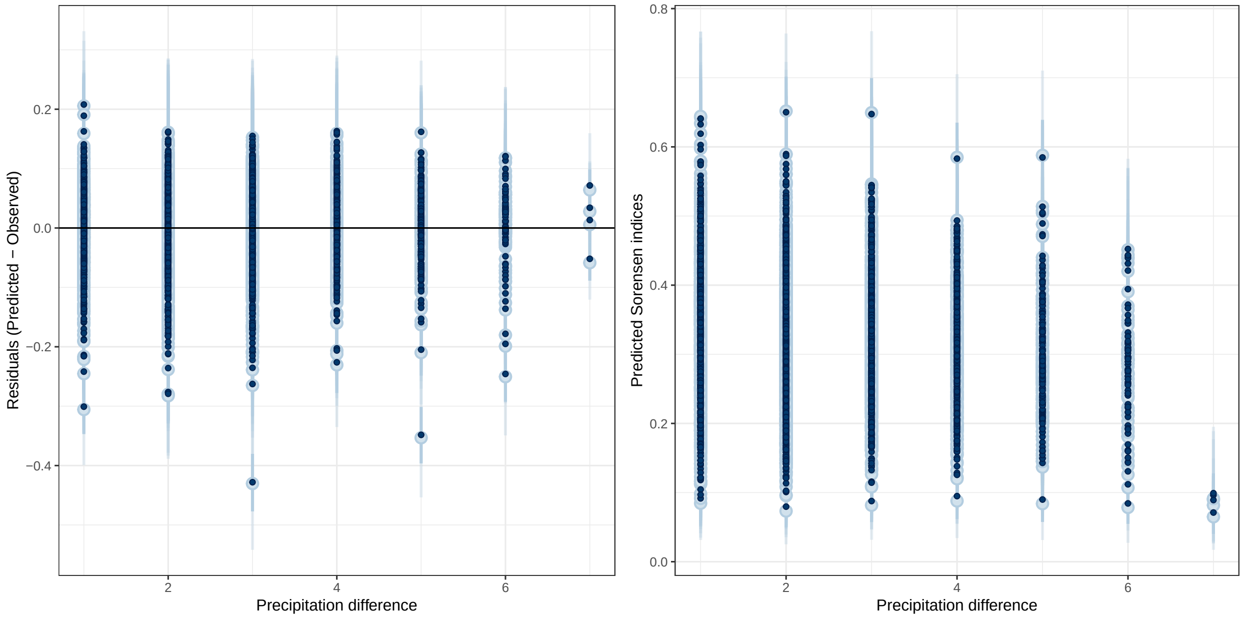

5.5.2.5 Residuals and predicted Sorenson indices against basin intercepts This plot shows 1) Residuals (dark blue points) and its distribution (light blue lines) plotted against basin intercepts, and 2) Predicted Sorenson indices (dark blue points) and its distribution (light blue lines) against basin intercepts.

```
grid.arrange(
  ppc_intervals(res_mean,residuals,basin_idx)+labs(y="Residuals (Predicted - Observed)",
                                                    x="Basin intercepts")+
    theme_bw()+hline_0()+legend_none(),
  ppc_intervals(sor_mean_pred,sor_pred,basin_idx)+labs(y="Predicted Sorensen indices",
                                                          x="Basin intercepts")+
    theme_bw()+legend_none(),
  ncol=2)
```

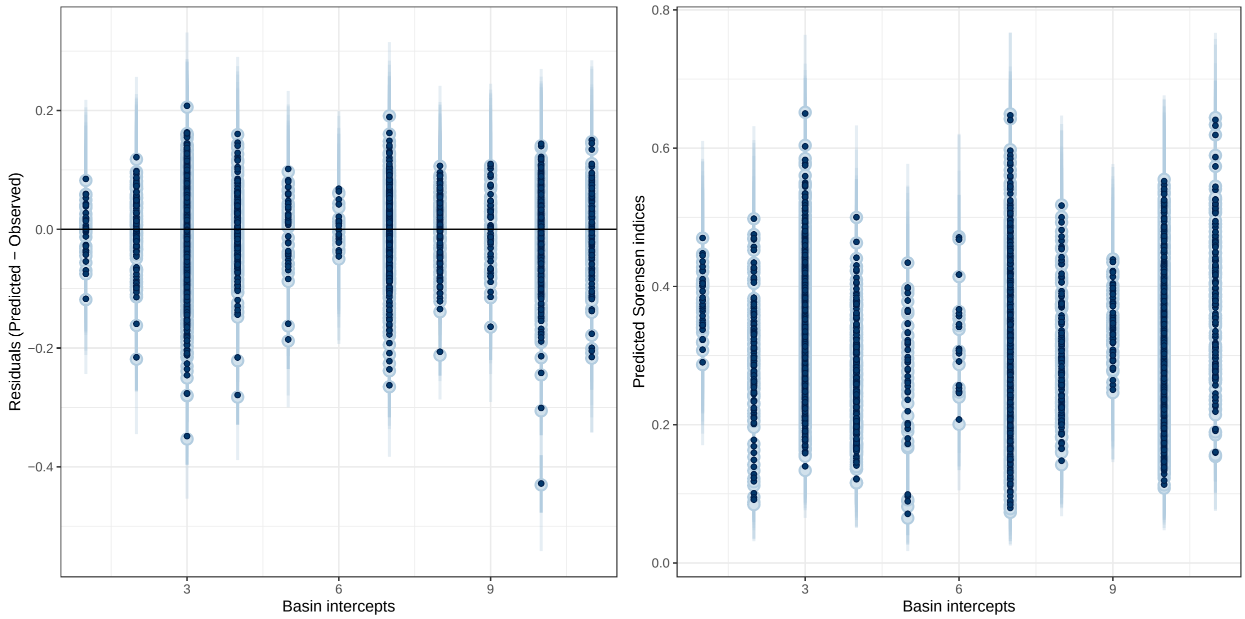

We see no systematic deviations.

5.5.2.6 Residuals and predicted Sorensen indices against sample intercepts This plot shows 1) Residuals (dark blue points) and its distribution (light blue lines) plotted against basin sample intercepts (1 and 2), and 2) Predicted Sorenson indices (dark blue points) and its distribution (light blue lines) against sample intercepts (1 and 2).

```
grid.arrange(
  ppc_intervals(res_mean,residuals,sample_idx1)+labs(y="Residuals (Predicted - Observed)",
                                                      x="Sample intercept 1")+
    theme_bw()+hline_0()+legend_none(),
  ppc_intervals(sor_mean_pred,sor_pred,sample_idx1)+labs(y="Predicted Sorenson indices",
                                                          x="Sample intercept 1")+
     theme_bw()+legend_none(),
  ncol=2)
```

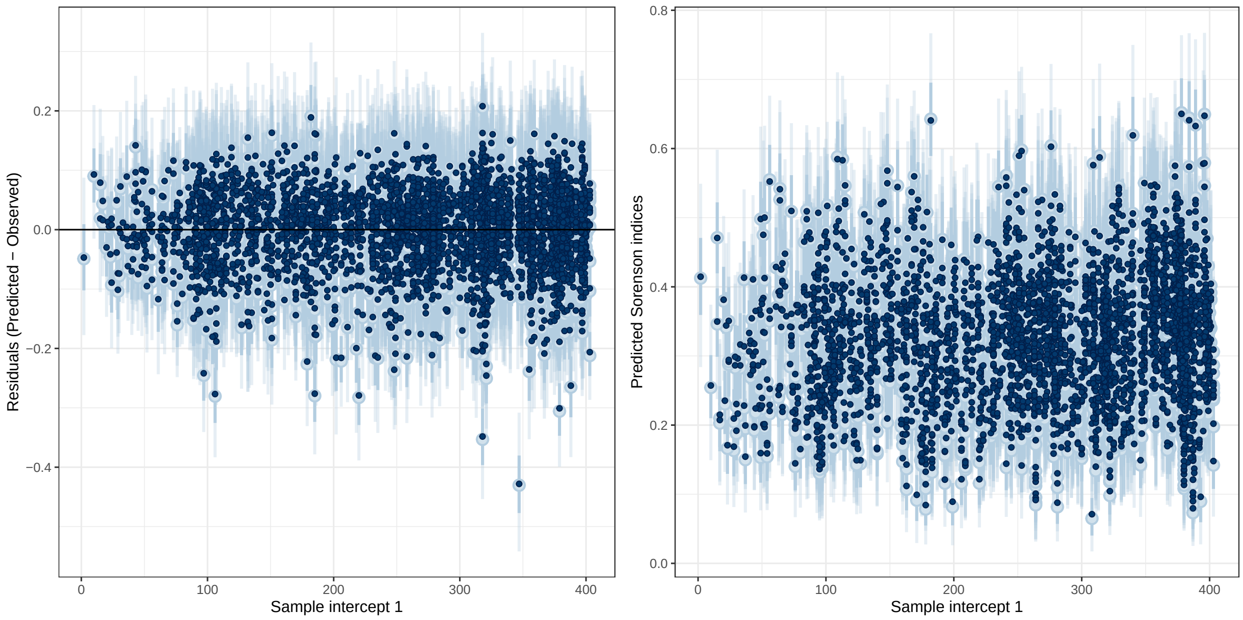


```
grid.arrange(
  ppc_intervals(res_mean,residuals,sample_idx2)+labs(y="Residuals (Predicted - Observed)",
                                                     x="Sample intercept 2")+
    theme_bw()+hline_0()+legend_none(),
  ppc_intervals(sor_mean_pred,sor_pred,sample_idx2)+labs(y="Predicted Sorenson indices",
                                                     x="Sample intercept 2")+
     theme_bw()+legend_none(),
  ncol=2)
```

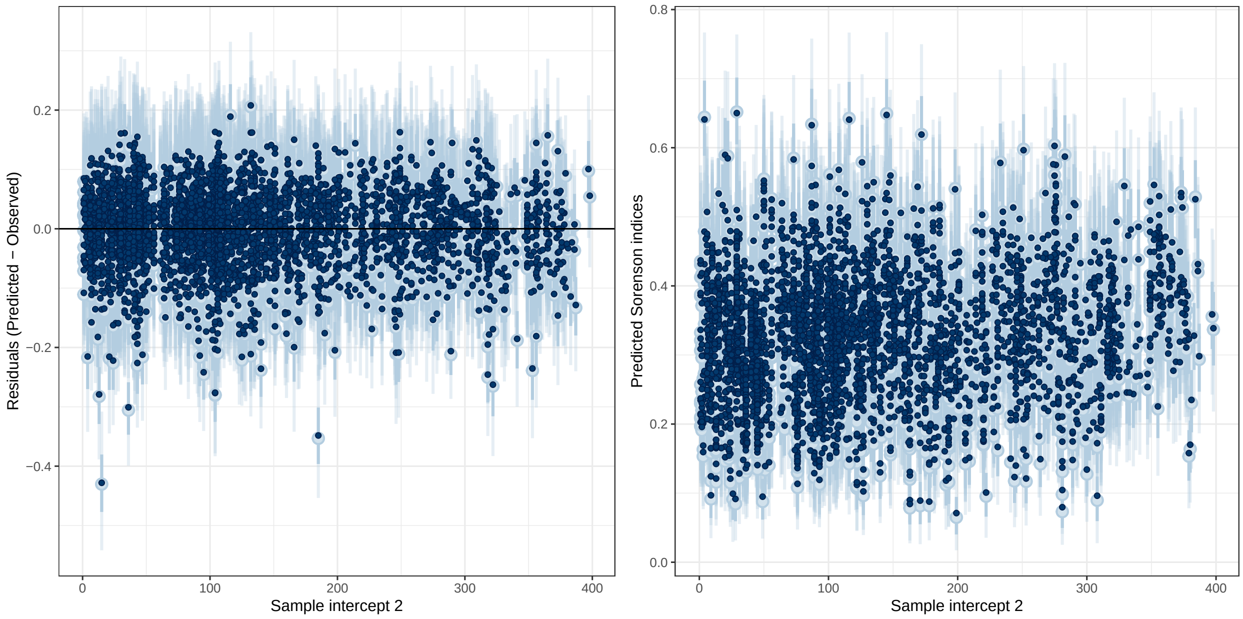

We see no systematic deviations.

5.6 Parameter estimates

```
posterior<-as.array(fit,pars=c("bnet[1]","bnet[2]","bnet[3]",
```

```
                              "bnet[4]","bnet[5]",
                              "bnet[6]",
                              "bnet[7]","bnet[8]",
                              "bnet[9]","bnet[10]",
                              "bnet[11]","mu_bnet"))
plot_title <- ggtitle("Network distance slopes")
mcmc_intervals(posterior,prob = 0.95) + plot_title+
  scale_y_discrete(labels=c("Ave","Cávado","Douro","Guadiana","Minho","Mira",
                           "Mondego",
                           "Sado","Sorraia","Tejo","Vouga","mu")) + vline_0()+
theme_bw()+theme(axis.text=element_text(size=14))+labs(x="Parameter estimate")
```

5.6.1 Network distance slopes

```
## Scale for 'y' is already present. Adding another scale for 'y', which will
## replace the existing scale.
```

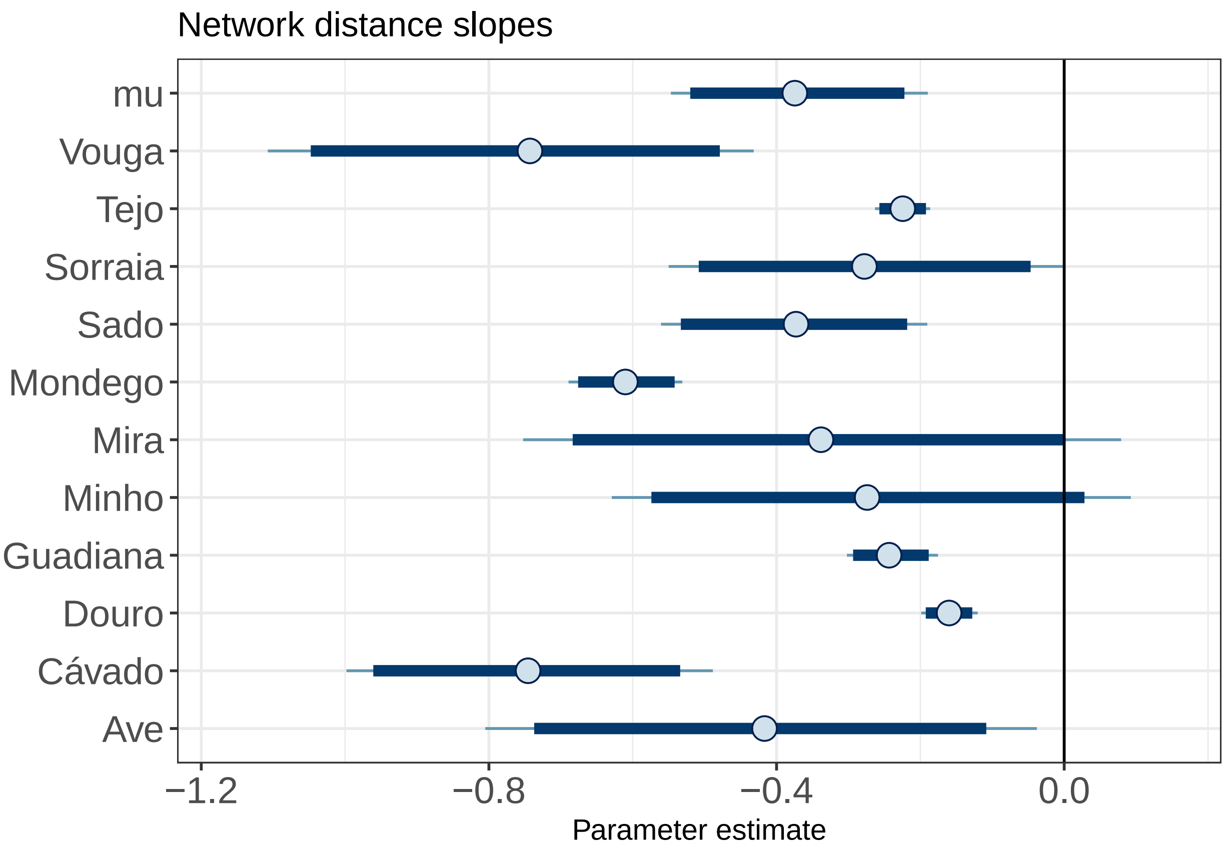


```
posterior<-as.array(fit,pars=c("bflow[1]","bflow[2]","bflow[3]",
                              "bflow[4]","bflow[5]",
                              "bflow[6]",
                              "bflow[7]","bflow[8]",
                              "bflow[9]","bflow[10]",
                              "bflow[11]","mu_bflow"))
plot_title <- ggtitle("Flow connection slopes")
mcmc_intervals(posterior,prob = 0.95) + plot_title+
```

```
  scale_y_discrete(labels=c("Ave","Cávado","Douro","Guadiana","Minho","Mira",
                            "Mondego",
                            "Sado","Sorraia","Tejo","Vouga","\u03BC")) + vline_0()+
theme_bw()+theme(axis.text=element_text(size=14))+labs(x="Parameter estimate")
```

5.6.2 Flow connection

```
## Scale for 'y' is already present. Adding another scale for 'y', which will
## replace the existing scale.
```

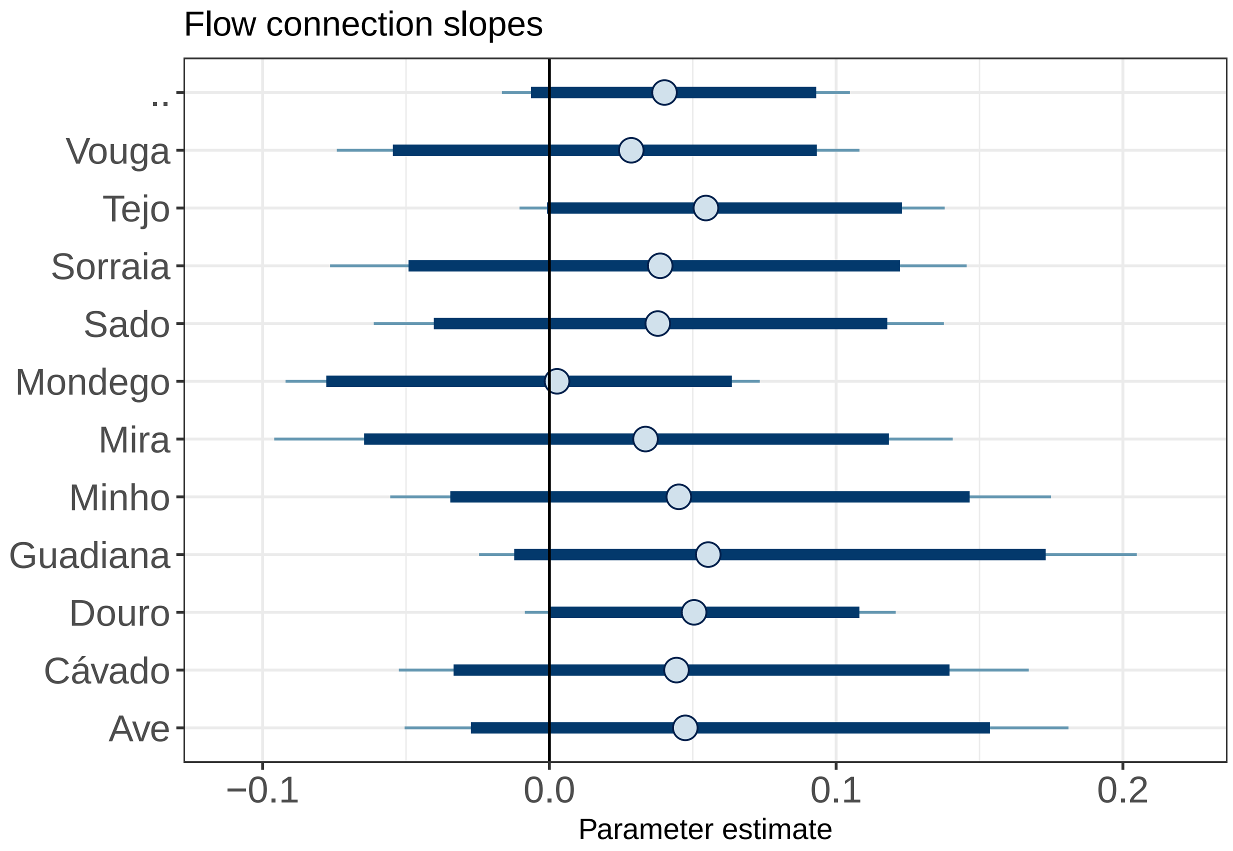


```
posterior<-as.array(fit,pars=c("bpp[1]","bpp[2]","bpp[3]",
                               "bpp[4]","bpp[5]",
                               "bpp[6]",
                               "bpp[7]","bpp[8]",
                               "bpp[9]","bpp[10]",
                               "bpp[11]","mu_bpp"))
plot_title <- ggtitle("Precipitation difference slopes")
mcmc_intervals(posterior,prob = 0.95) + plot_title+
  scale_y_discrete(labels=c("Ave","Cávado","Douro","Guadiana","Minho","Mira",
                            "Mondego",
                            "Sado","Sorraia","Tejo","Vouga","\u03BC")) + vline_0()+
theme_bw()+theme(axis.text=element_text(size=14))+labs(x="Parameter estimate")
```

5.6.3 Precipitation difference

```
## Scale for 'y' is already present. Adding another scale for 'y', which will
```

```
## replace the existing scale.
```

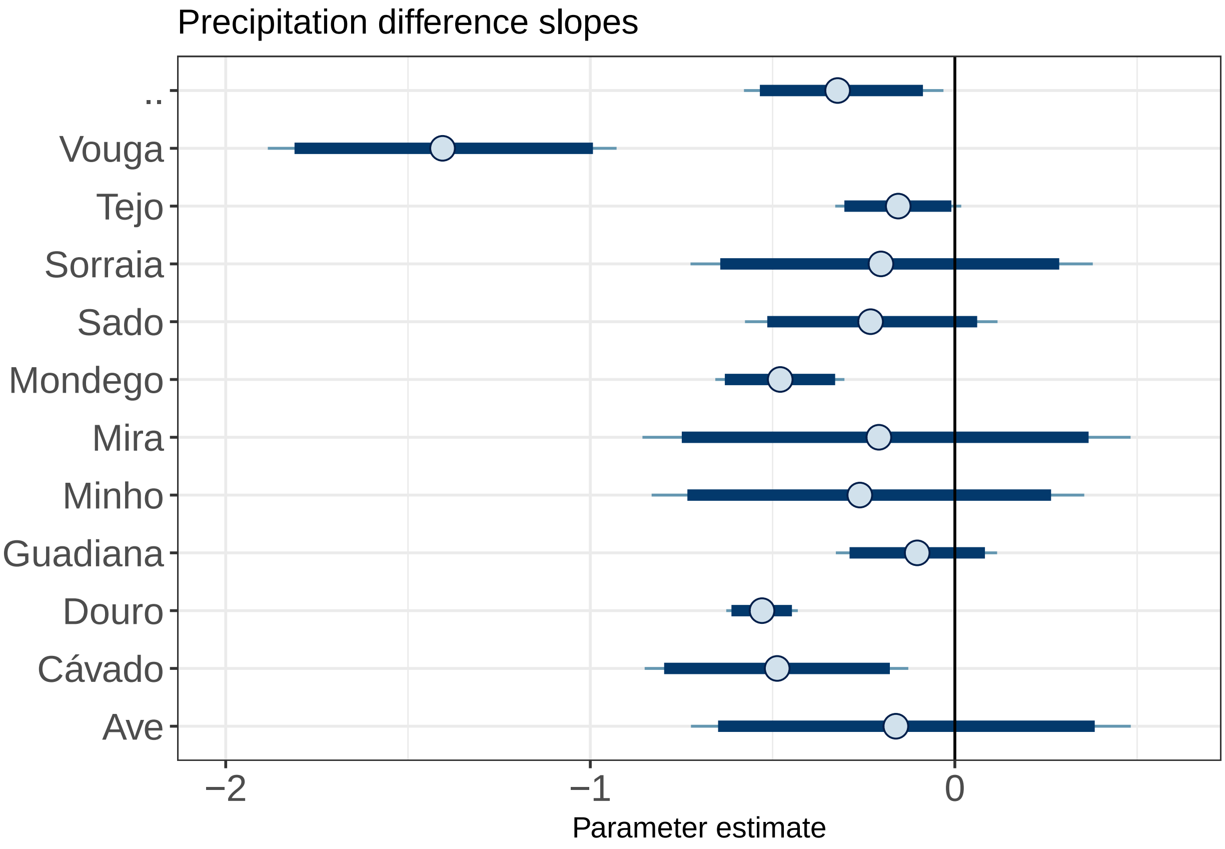


```
posterior<-as.array(fit,pars=c("a_strahler[1]","a_strahler[2]","a_strahler[3]",
                               "a_strahler[4]","a_strahler[5]","a_strahler[6]",
                               "a_strahler[7]"))
plot_title <- ggtitle("Strahler order difference slopes")
mcmc_intervals(posterior,prob = 0.95) + plot_title+
  scale_y_discrete(labels=c("0","1","2","3","4","5","6")) + vline_0()+
theme_bw()+theme(axis.text=element_text(size=14))+labs(x="Parameter estimate")
```

5.6.4 Strahler order difference

```
## Scale for 'y' is already present. Adding another scale for 'y', which will
## replace the existing scale.
```

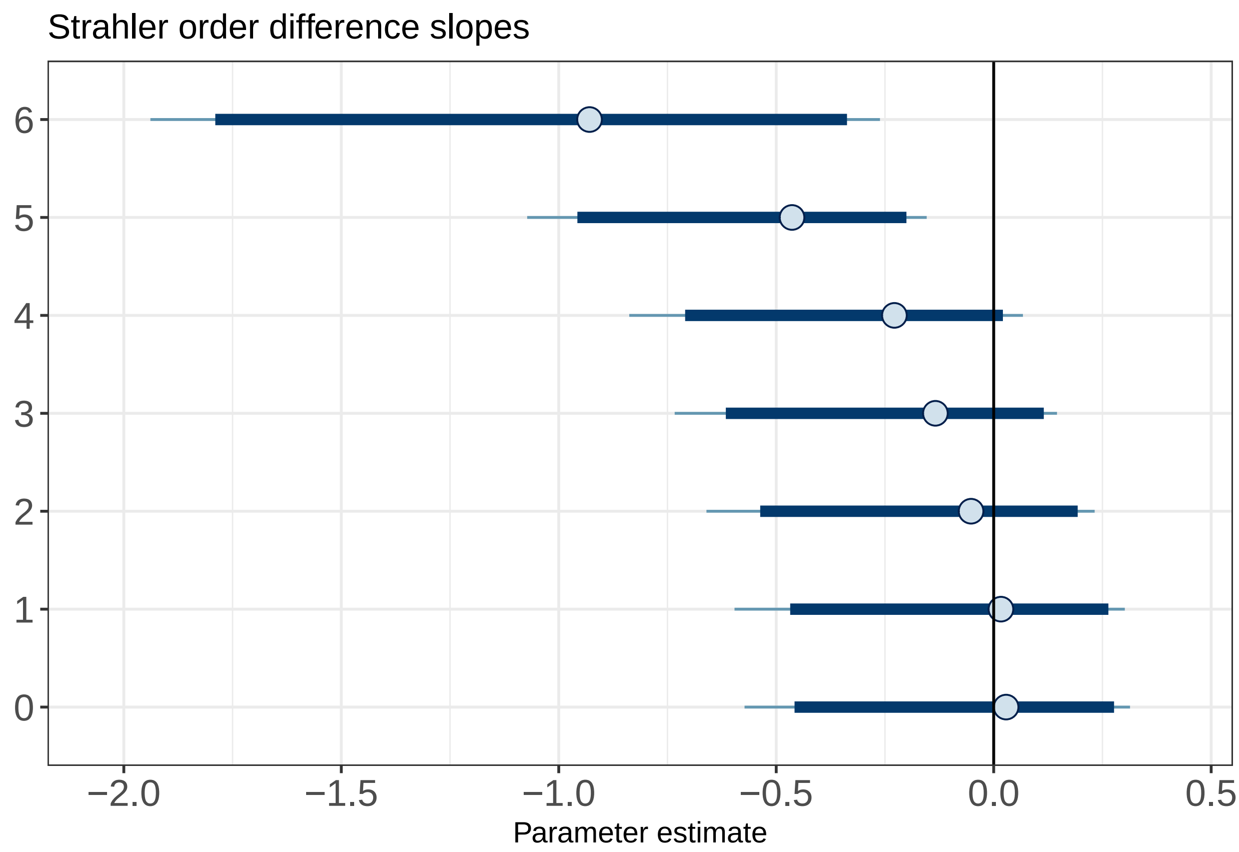
Strahler order difference slopes
6
5
4
3
2
1
0
−2.0
−1.5
−1.0
−0.5
0.0
0.5
Parameter estimate
